# Strong Ligand Stabilization Based on π‐Extension in a Series of Ruthenium Terpyridine Water Oxidation Catalysts

**DOI:** 10.1002/chem.202102905

**Published:** 2021-11-11

**Authors:** Sebastian Amthor, David Hernández‐Castillo, Boris Maryasin, Phillip Seeber, Alexander K. Mengele, Stefanie Gräfe, Leticia González, Sven Rau

**Affiliations:** ^1^ Inorganic Chemistry I Ulm University Albert-Einstein-Allee 11 89081 Ulm Germany; ^2^ Institute of Theoretical Chemistry Faculty of Chemistry University of Vienna Währinger Str. 17 1090 Vienna Austria; ^3^ Institute of Organic Chemistry, Faculty of Chemistry University of Vienna Währinger Str. 38 1090 Vienna Austria; ^4^ Institute of Physical Chemistry and Abbe Center of Photonics Friedrich-Schiller University Jena Helmholtzweg 4 07743 Jena Germany

**Keywords:** DFT calculations, homogenous catalysis, ligand, ruthenium-terpyridine complex exchange, water oxidation

## Abstract

The substitution behavior of the monodentate Cl ligand of a series of ruthenium(II) terpyridine complexes (terpyridine (tpy)=2,2′:6′,2′′‐terpyridine) has been investigated. ^1^H NMR kinetic experiments of the dissociation of the chloro ligand in D_2_O for the complexes [Ru(tpy)(bpy)Cl]Cl (**1**, bpy=2,2’‐bipyridine) and [Ru(tpy)(dppz)Cl]Cl (**2**, dppz=dipyrido[3,2‐*a*:2′,3′‐*c*]phenazine) as well as the binuclear complex [Ru(bpy)_2_(tpphz)Ru(tpy)Cl]Cl_3_ (**3** **b**, tpphz=tetrapyrido[3,2‐*a*:2′,3′‐*c*:3′′,2′′‐*h*:2′′′,3′′′‐*j*]phenazine) were conducted, showing increased stability of the chloride ligand for compounds **2** and **3** due to the extended π‐system. Compounds **1**–**5** (**4**=[Ru(tbbpy)_2_(tpphz)Ru(tpy)Cl](PF_6_)_3_, **5**=[Ru(bpy)_2_(tpphz)Ru(tpy)(C_3_H_8_OS)/(H_2_O)](PF_6_)_3_, tbbpy=4,4′‐di‐*tert*‐butyl‐2,2′‐bipyridine) are tested for their ability to run water oxidation catalysis (WOC) using cerium(IV) as sacrificial oxidant. The WOC experiments suggest that the stability of monodentate (chloride) ligand strongly correlates to catalytic performance, which follows the trend **1**>**2**>**5**≥**3**>**4**. This is also substantiated by quantum chemical calculations, which indicate a stronger binding for the chloride ligand based on the extended π‐systems in compounds **2** and **3**. Additionally, a theoretical model of the mechanism of the oxygen evolution of compounds **1** and **2** is presented; this suggests no differences in the elementary steps of the catalytic cycle within the bpy to the dppz complex, thus suggesting that differences in the catalytic performance are indeed based on ligand stability. Due to the presence of a photosensitizer and a catalytic unit, binuclear complexes **3** and **4** were tested for photocatalytic water oxidation. The bridging ligand architecture, however, inhibits the effective electron‐transfer cascade that would allow photocatalysis to run efficiently. The findings of this study can elucidate critical factors in catalyst design.

## Graphical abstract

**The correlation** between the stability of a monodentate chloride ligand and the catalytic water oxidation performance of a set of ruthenium(II) terpyridine complexes based on π‐extension of a bipyridine is investigated. The findings are supported by theoretical calculations and show a significant dependence of the stability of the chloride ligand on the π‐extension and, hence, a decrease in the catalytic turnover number and turnover frequency.

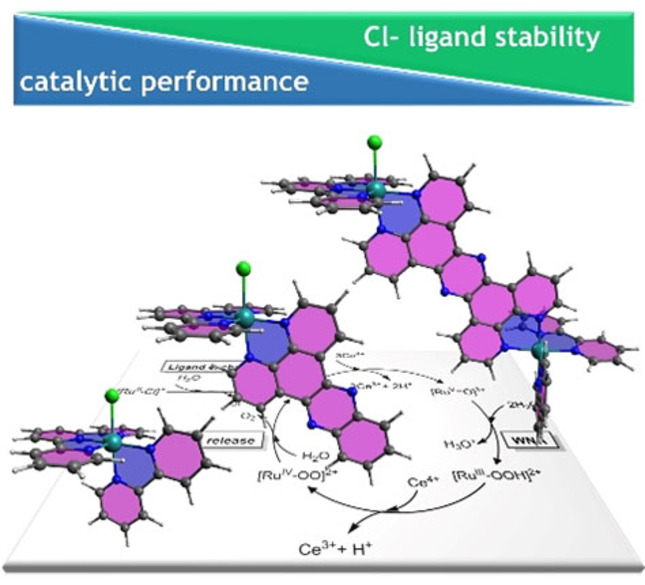

## Introduction

With the menace of climate change driven by our increasing energy demand and the associated combustion of ever‐increasing amounts of fossil fuels overshadowing our daily lives, new approaches towards a sustainable society have to be developed. Generally, most technologies being developed make use of the sun as energy source, as it is inexhaustible and ubiquitously available. The light energy emitted by the sun can be collected with solar cells and stored either in batteries or in chemical bonds by solar fuel production. Recent advances in solar cell development have increased the efficiency of different types of solar cells including perovskite,[^1^, ^2^, ^3^] dye‐sensitized,[^4^, ^5^, ^6^] and polymer‐based cells.[^7^, ^8^] However, energy conversion is not the critical factor in the equation. Because the light energy harvested by solar cells has to be distributed or stored, the limiting factor of renewable energies are the efficiencies at which this energy can be stored and converted back to electricity. Although recent developments in battery research showed huge improvements in efficiencies and stability,[^9^, ^10^, ^11^] the production of solar fuels for large‐scale application is appealing because, to a certain extent, the infrastructure in place for fossil fuels can be utilized for distribution. Additionally, chemical bonds are the cheapest and most stable way to store the light energy when compared to batteries or pumped hydroelectricity. Using water as the source of electrons and protons resembles a sustainable way of fuel production. The two key steps being water oxidation and proton reduction.^[12]^ While the hydrogen evolution reaction directly delivers hydrogen, which can be used as a fuel, water oxidation catalysis (WOC) has to supply the electrons for this reaction. If water can be used as an electron source, water splitting can proceed sustainably. However, water oxidation is considered to be the more complex reaction, as it involves four consecutive electron‐transfer processes.^[13]^ In general the reaction may proceed heterogeneously or homogenously. In the former case, mainly metal oxide particles, for example, TiO_2_[^14^, ^15^] CoO_*x*_,[^16^, ^17^] Ni(OH)_2_ ^[18]^ or FeNiO_*x*_,^[19]^ or surfaces are used. In the latter case, molecular catalysts, mostly transition metal complexes of nickel,^[20]^ copper,[^21^, ^22^] iridium,[^23^, ^24^] and ruthenium[^25^, ^26^, ^27^] are employed to facilitate the catalysis. The advantage of using molecular metal complexes over surfaces or particles is that in the case of a molecule the overall activity per metal atom is higher. This can easily be understood when considering that in particles and surfaces most of the metal atoms are situated in the bulk and not accessible to the substrate on the surface. Another advantage of molecular catalysts is that their catalytic behavior in many cases can be influenced by substitution of the molecule by different groups, which can increase selectivity and activity as well as stability of the system.^[28]^ However, tuning the selectivity, activity and stability of molecular catalysts can only be successful if the underlying mechanisms are known.^[21]^ A class of well‐established molecular catalysts, which can facilitate the complex reaction of water oxidation, are ruthenium(II) complexes of either 2,2′:6′,2′′‐terpyridine (tpy),^[29]^ 2,9‐di(pyridin‐2‐yl)‐1,10‐phenanthroline (dpp) or [2,2′‐bipyridine]‐6,6′‐dicarboxylic acid (bda). The mechanism for Ru(dpp)^[30]^ and Ru(bda) proceeds via the formation of a seventh coordination site.^[31]^ In contrast, Ru(tpy)‐based water oxidation catalysis is considered to go hand in hand with loss of the monodentate ligand (e. g., Cl^−^).^[32]^ This substitution of the monodentate ligand by water or the hydroxy‐moiety of an alcohol is considered a key step in water or alcohol oxidation.[^29^, ^32^] Normally this substitution proceeds slowly in water and can be accelerated using silver‐containing salts (e. g., AgNO_3_ or AgClO_4_).[^29^, ^33^] If this substitution is run in water without addition of silver salts, the substitution of a chloro to an aquo ligand proceeds extremely slowly.^[29]^ However, this activation step is crucial for the kinetics of the oxygen evolution.^[32]^ Therefore, in order to build up more complex systems or embed these catalysts into solid‐state materials, a deep understanding of the stabilization of the monodentate ligand, and thus, the activation of the catalysts, is mandatory. In this work we investigated the effect of π‐extended ligands on the substitution of a chloride ligand in a series of Ru(tpy) catalysts to elucidate potential ways to form molecular dyads capable of light‐driven water oxidation.

## Results and Discussion

### Synthesis and characterization

**Figure 1:**
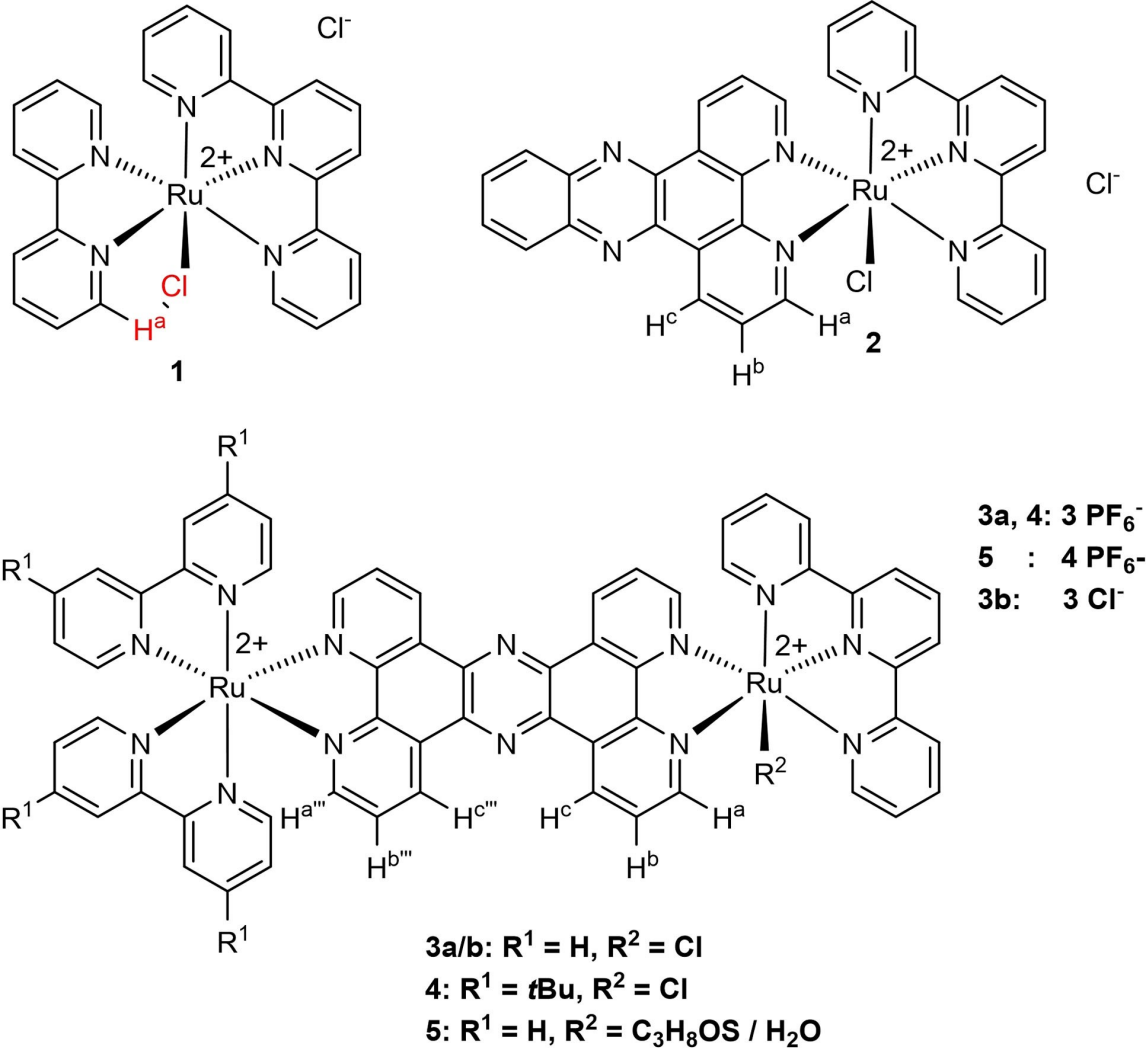
Complexes investigated in this study.

**Figure 2:**
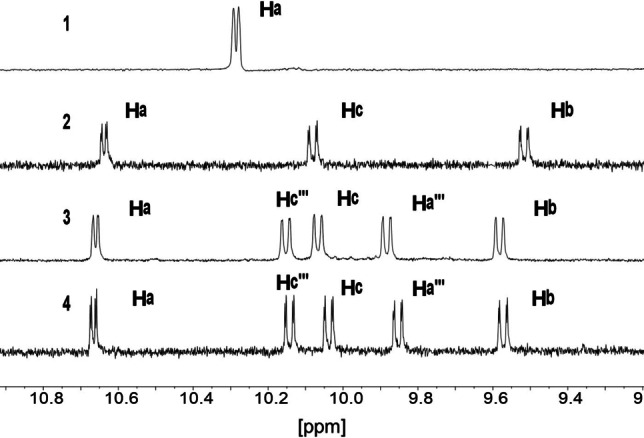
^1^H NMR spectra of the protons, which will be shifted strongly upon chloride substitution (signals in close proximity to the monodentate ligand, i. e., H^a^,H^c^; Figure 1) in the aromatic region of compound **1**–**4** in an [D_6_]acetone/[D_4_]methanol mixture (9 : 1, *v*/*v*) at 25 °C. Note that the spectra for **3** **a** and **3** **b** are identical.

A set of ruthenium terpyridine model complexes bearing a 2,2′‐bipyridine (**1**) and a dppz (**2**) ligand were synthesized in a microwave‐assisted reaction of [Ru(tpy)Cl_3_] with the respective ligand in a dimethylformamide/triethylamine mixture by a known procedure.^[33]^ Additionally, the binuclear complexes **3** and **4** were synthesized by reaction of [Ru(R_2_bpy)_2_(tpphz)]Cl_2_ with [Ru(tpy)Cl_3_] in a complex as ligand approach (the [Ru(R_2_bpy)_2_(tpphz)]Cl_2_ here acts as the ligand for [Ru(tpy)Cl_3_]) in an ethanol water mixture for 24 h (Figure 1). Replacement of the chloride ligand in **3** by 2‐(methylthio)ethan‐1‐ol in an ethanol water mixture yielded compound **5**. Compounds **1**–**4** were characterized by ^1^H NMR and UV‐vis spectroscopy as well as mass spectrometry (see Figures S1–S13 in the Supporting Information). The peak assignment of the ^1^H NMR spectra was done by literature reported spectra of similar dinuclear RuPt, RuPd and RuRh complexes.[^34^, ^35^] Figure 2 shows the peak region, which is most influenced by the nature of the monodentate ligand. Compound **2** features a significant deshielding of the H^a^ proton of 0.4 ppm compared to **1** due to the introduction of the electron‐withdrawing phenazine sphere in the dppz ligand. Compound **3** and **4** show similar signal shifts, introduction of the second coordination sphere and the second metal center does not significantly alter the peak positions of H^a^–H^c^ compared to **2**.

**Figure 3:**
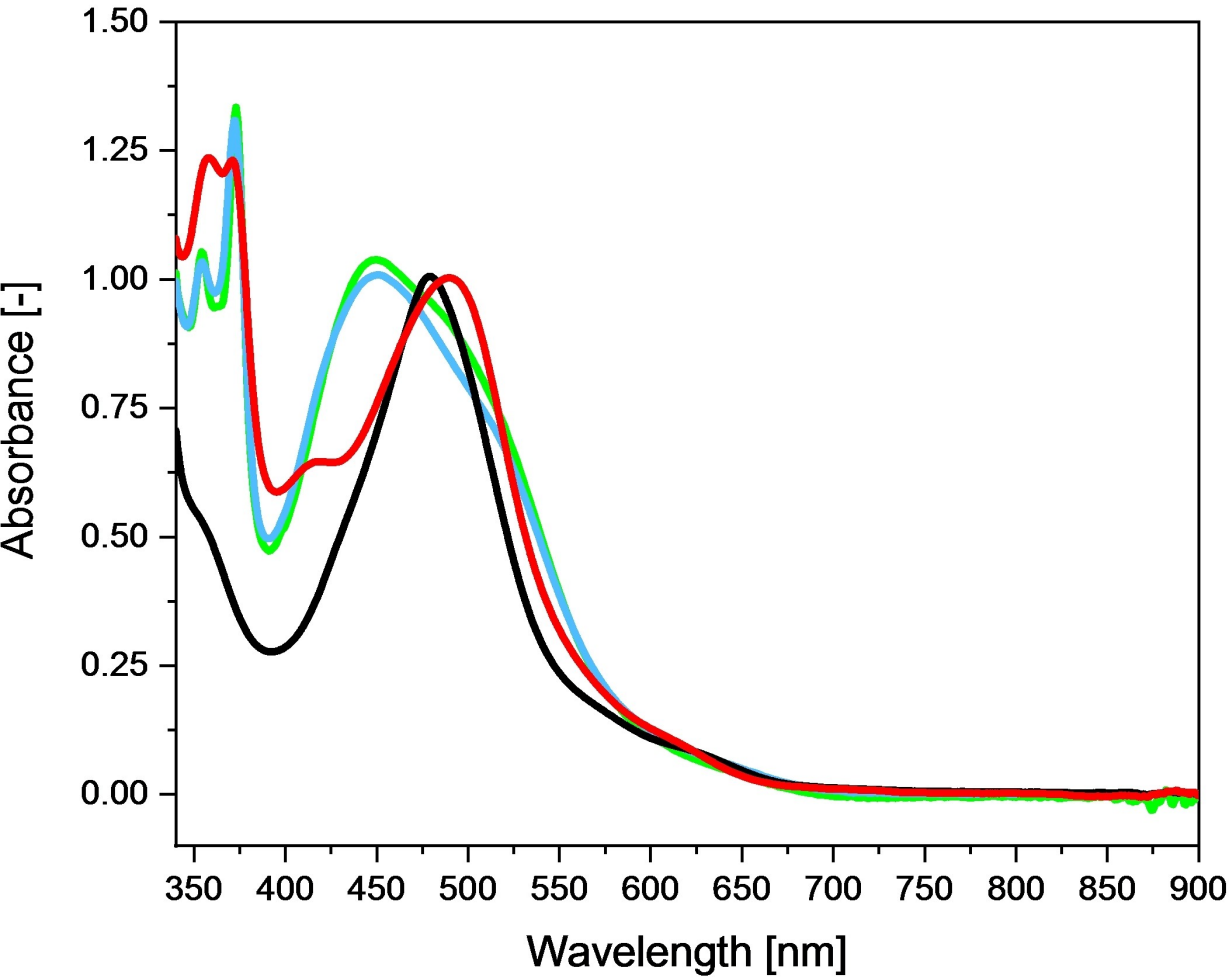
Normalized (to maximum absorbance of **1** at 478 nm) UV‐vis absorption spectra of compound **1** (black), **2** (red), **3** (blue), **4** (green).

Figure 3 compares the UV‐vis absorption spectra of compound **1**–**4**. The spectral features are summarized in Table 1. Upon changing the bpy ligand to a dppz ligand with a larger π‐system from **1** to **2**, the absorption maximum of the spin‐allowed metal‐to‐ligand charge‐transfer ^1^MLCT transition shifts from 480 nm to 490 nm.^[29]^ Due to the phenazine sphere in **2**, also the characteristic ligand‐centered band (LC) at around 380 nm can be observed.[^35^, ^36^] The dinuclear complexes **3** and **4** show a broad absorption peak from 400–450 nm with a maximum at 450 nm, which can be attributed to the ^1^MLCT band of the [Ru(bpy)_2_(tpphz)] sphere[^35^, ^36^] The shoulder located at 550 nm nm can be assigned to the [Ru(tpy)(tpphz)Cl] moiety of the complex. Emission spectroscopy for compound **3** and **4** shows no observable emission for compound **3** and very weak (within the Raman bands) emission for compound **4** indicating that quenching of the emission of the [Ru(R_2_bpy)_2_(tpphz)] moiety by the [Ru(tpy)(tpphz)Cl] is very efficient (data not shown).

**Table 1:** UV‐vis spectroscopic and electrochemical data for compounds **1**–**6**.

	*λ*_max_ [nm]			Emission	$E_{1/2}^{ox}$ [V vs.
entry	phenazine	cat.	PS		Ag/AgCl]
**1^a^**	/	478	/	/	0.36^[d]^, 0.71^[c]^
**2^a^**	357, 370	491	/	/	0.40^[d]^
**3^b^**	353, 372	450–700	450	617	0.55, 1.05^[d]^
**4^b^**	353, 372	450–650	450	/	0.46, 0.84^[d]^

[a]: measured in H_2_O; [b]: measured in acetonitrile; [c]: H_2_O with KCl (0.1 M) supporting electrolyte; [d]: acetonitrile with TBAPF_6_ (0.1 M) supporting electrolyte vs. Fc/Fc^+^; compound **3** here refers to both **3** **a** and **3** **b**, as these exhibit similar properties.

Electrochemical characterization also revealed that there is a significant influence of the substituents on the bipyridine ligand (H vs. *t*Bu) to the oxidation potential Ru^II^/Ru^III^ on both the [Ru(R_2_bpy)_2_(tpphz)] (shift of ca. 200 mV) and the [Ru(tpy)(tpphz)Cl] (shift of ca. 100 mV) moiety of the complex (Table 1). This fact illustrates the electronic communication of both moieties with each other as well as the electron donating nature of the *t*Bu substituents. This is also highlighted by a shift in the reduction potential of the phenazine moiety of the tpphz ligand from −1.09 V in **3** to −1.23 V in **4**. Moreover, compared to compounds **1** and **2**, the oxidation potential of the catalytic unit in the binuclear compounds **3** and **4** is shifted anodically. The effect most probably stems from the electron‐withdrawing nature of the second metal center towards the phenazine moiety. In order to evaluate the influence of the altered ligand environment on the oxidation potentials relevant to catalysis, the attempt was made to resolve also higher oxidation states of the ruthenium center coordinated to the terpyridine ligand. As obtained by differential pulse voltammetry, the electron density on the bidentate ligand seems to influences the potential of the Ru^IV^/Ru^III^ wave (Figure S16). The potential is anodically shifted upon introduction of a dppz ligand, manifesting in a shift of the potential of the Ru^IV^/Ru^III^ wave from 0.64 V versus Fc^+^/Fc^0^ in **1** to 0.93 versus Fc^+^/Fc^0^ in **2**. For binuclear complexes **3** **a** and **4**, the waves cannot easily be evaluated due to overlap of waves attributed to both of the metal centers. It seems to be shifted even more anodically for compound **4** to 1.22 V versus Fc^+^/Fc^0^. For compound **3** **a**, no assessment of the wave was possible. However, these results indicate, that the driving force for water oxidation of the system is also altered by extension of the π‐system and hence an effect on the turnover numbers (TONs) cannot be excluded. Nevertheless, theoretical calculations imply that the general mechanism and associated energies are not changed in the aquo coordinated Ru(tpy) moieties irrespective of the actual bidentate ligand (see Mechanistic Calculations section). It has to be noted, that the described experiments were conducted in the absence of water and for the chloride coordinated original complexes. The situation might drastically change upon substitution of the chloride ligand with an aquo ligand in aqueous media.

### Substitution kinetics of the chloride ligand

**Figure 4:**
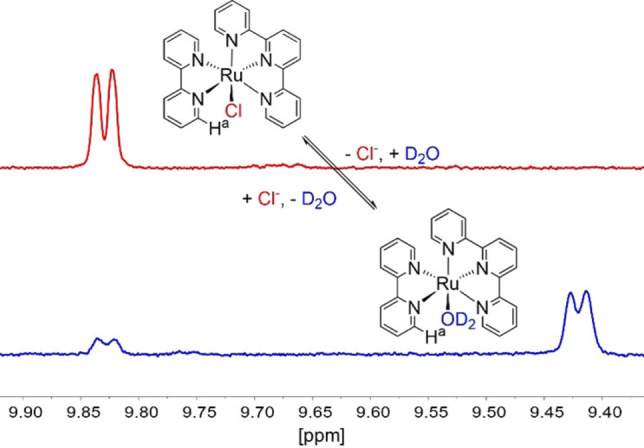
^1^H NMR spectra of compound **1** in D_2_O at 25 °C, top: freshly prepared solution, bottom: solution after 24 h.

**Figure 5:**
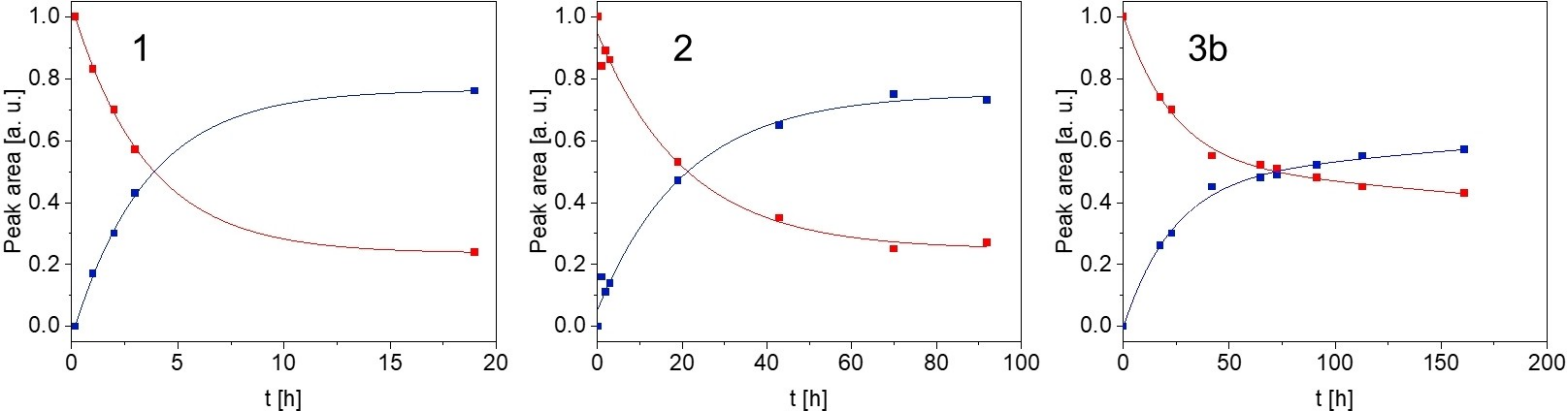
Peak area of H^a^ vs. time for the RuCl (red) and the Ru(D_2_O) (blue) species derived from ^1^H NMR spectra of compound **1**, **2** and **3** **b** taken after different times in D_2_O at 25 °C.

Based on the structure of the [Ru(tpy)Cl] moiety, the chloride ligand lies in the same plane as the bidentate ligand (e. g., bpy) and therefore the H^a^ signal is strongly influenced by the nature of the monodentate ligand. The characteristic shift of the H^a^‐proton (indicated in Figure 4) can therefore be used as a sensitive probe for substitution.^[29]^ Alternation of the bidentate ligand to a π‐conjugated system like dppz considerably slows down the substitution of the chloro‐ligand in D_2_O for compound **2**, as can be seen from kinetic NMR measurements (Figure 5). The half‐life for the RuCl species derived from the ^1^H NMR data by an exponential fit yield values of 4 and 21 h for **1** and **2**, respectively, showing a fivefold increase by π‐extension in the bidentate ligand from **1** to **2**. Compound **3** **a** also exhibits considerably higher stability for the chloro ligand. For the binuclear complex **3** **a** the substitution is very slow, correlated to a half‐life of the RuCl species of ∼72 h (18‐fold increase compared to **1**); Figure 5. The equilibrium constants (*K*_e_) derived from the ^1^H NMR kinetic experiments show values of 3.2, 2.7 and 1.3 for **1**, **2** and **3** **b**, respectively. As was suggested by other studies, replacement of this ligand has to be done prior to catalysis to render the systems active.[^29^, ^32^, ^37^]

### Catalysis

**Figure 6:**
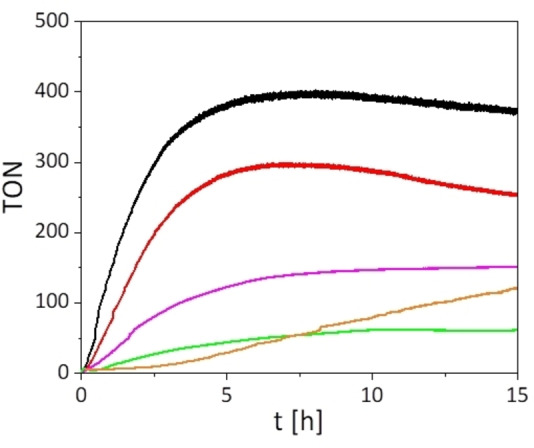
Catalytic TONs using cerium(IV)ammonium nitrate as sacrificial oxidant in water (**1**: black, **2**: red, **3** **b**: orange, **4**: green, **5**: purple). Conditions: **1** and **2**: cat: 100 μM, CAN: 0.5 M, H_2_O; **3**, **4** and **5**: cat: 70 μM, CAN: 0.35 M, H_2_O. (The best result for each catalyst is shown, errors are given in Table 2)

Compound **1**, **2** and **3** **b**, **4**, **5** were tested towards their capability of water oxidation using cerium(IV)ammonium nitrate (CAN) as sacrificial oxidant. To this end, the complexes were dissolved in water and CAN was added. The solution was stirred in the dark and oxygen evolution was detected with an in operando sensor setup developed in our group earlier.^[38]^ Experiments were conducted as doublets, the data is summarized in Table 2. All complexes were able to generate oxygen from water by chemical oxidation. The dppz‐derivative **2** generated a moderate TON of 300 after 20 h compared to the well investigated compound **1** with 400 after 20 h. This fact strongly supports the data acquired by the ^1^H NMR study investigating the kinetics of the chloride substitution and suggests that the stability of the ruthenium chloride bond limits the efficiency of WOC in compound **2**. Additionally, significantly lower TONs were observed for the binuclear complexes **3** **b** and **4** of 120 and 55, respectively, compared to **1** under identical conditions. Due to the even slower substitution of the chloride ligand in these systems, it was assumed that the substitution is limiting the activity of the catalyst. Therefore, replacing the chloro‐ligand in **3** **b** by a 2‐(methylthio)ethan‐1‐ol, which was immediately replaced by an aquo‐ligand within the catalytic mixture, was done prior to catalysis. As a result, the TON almost doubled from 80 to 150 after 10 h (**3** **b** vs. **5**, Figure 6). While **3** **b** and **5** showed a similar TON of about 150 at the plateau, the turnover frequency (TOF) significantly increased by prior removal of the monodentate chloride ligand in compound **5** (Figure 6). This behavior further supports the fact that blocking of the catalytic site by a very well stabilized chloro‐ligand in **2**, **3** **b** and **4** is responsible for the poor catalytic performance. Due to slower substitution of the Cl ligand, the active aquo‐species [Ru(tpy)(L)(H_2_O)] (L=bpy, dppz or tpphz) in solution is formed over a longer period of time, limiting catalysis in the initial phase, as compared to **1**. As compounds **3** and **4** exhibit a catalytic and a sensitizer moiety, their ability towards photocatalytic water oxidation was tested. Both systems are incapable of oxidizing water photochemically (Figures S17 and S18). This indicates that the bridging ligand architecture might not allow the necessary electron‐transfer cascade to enable WOC. However, this might not be true for an intermolecular system in which photocatalysis might still be observed.

**Table 2:** TONs of compound **1**, **3**, **6** and **7** with (NH_4_)_2_[Ce(NO_3_)_6_] as the sacrificial oxidant measured in this study and comparison to literature values.

		Turnover				
TON	**1** (lit.)	**1**	**2**	**3** **b**	**4**	**5**
Ce^IV^	390^[13]^	400±4 (5 h)	300±66 (5 h)	175±40 (21 h)	57±26 (10 h)	150±31 (10 h)
[Ru(bpy)_3_]^2+^	1.4^[23]^	n.d.	n.d.	n.d.	n.d.	n.d.

### Mechanistic calculations

**Figure 7:**
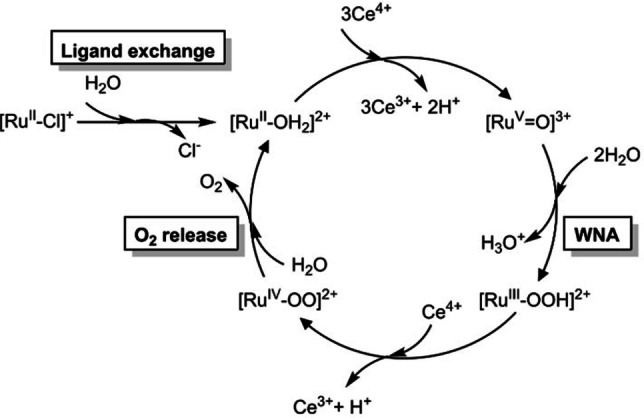
General catalytic cycle of the water oxidation reaction in mononuclear Ruthenium complexes.

**Figure 8:**
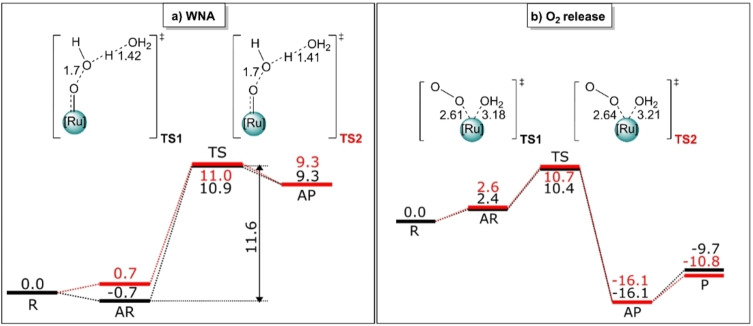
Reaction mechanism and computed relative Gibbs free energies (Δ*G* ${}_{298,H{}_{2}O}$ [kcal/mol]) for the a) water nucleophilic attack (WNA) and b) O_2_‐release events with corresponding reactants (R), associated reactants (AR), transition states (TS), associated products (AP) and product (P) for complex **1** (black) and **2** (red). In (a), the energy of the product (P) is not shown because the sum of the energies of separated products corresponds to the unphysical result: the product is energetically higher than the corresponding transition states. The latter is due to the insufficient stabilization of the “naked” (without explicit solvent molecules) hydronium H_3_O^+^. The sum of the reactant energies is taken as a reference (0.0 kcal/mol). Level of theory: UPBE0‐D3BJ‐CPCM(water)/def2‐TZVP//UPBE0‐D3BJ‐CPCM(water)/def2‐SVP.

As shown above, the increase in size of the π ligand from bpy to dppz (going from complex **1** to **2**) considerably reduces the obtainable TON. Even though the ligand exchange step could decrease the overall efficiency of the catalytic behavior, a priori it is not possible to discard that the ligand alone could not affect the reaction barriers during the water oxidation reactions and, hence, be responsible of the experimental difference in the TON. Therefore, we used density functional theory (DFT) to assess the impact of the ligands on both the catalytic water oxidation cycle and the ligand exchange step. Based on previous studies on complex **1**[^39^, ^40^] and analogues like [Ru^II^(tpy)(bpm)(OH_2_)]^2+^ (bpm=2.2’‐bipyrimidine),[^41^, ^42^] we derived the catalytic cycle for the water oxidation including the induction step for the ligand exchange (Figure 7). Accordingly, the full reaction includes three steps. In the first, labelled as the “ligand exchange”, the active aquo catalyst [Ru^II^‐OH_2_]^2+^ is generated. Subsequently, the catalytic cycle starts with two proton‐coupled‐electron‐transfer (PCET) steps and an additional oxidation that leads to the [Ru^V^=O]^3+^ species. This is subject to the water nucleophilic attack step (labeled “WNA”), responsible for the important O−O bond formation that leads to [Ru^III^‐OOH]^2+^. After a subsequent PCET step, the final oxygen release (“O_2_ release” step) operates regenerating the active catalyst, thus completing the catalytic cycle. While the oxidation and PCET steps are considered barrierless,^[40]^ the WNA and O_2_ release steps involve a transition state and therefore an associated kinetic reaction barrier. Hence, depending on the ligands, WNA or the O_2_ release have the potential to be the rate limiting step. In order to elucidate, we computed the reaction barriers associated to both steps for complexes **1** and **2**. Figure 8 shows the reaction profiles including the formation of the associated reactants and associated products. For the WNA step (Figure 8a), the energy difference between the transition states and the isolated reactants with two explicit water molecules results in barriers of ΔG≠298,H2O of about 11.0 kcal/mol for both compounds **1** and **2**. Likewise, for the O_2_ release (Figure 8b), the calculations also predict nearly identical barriers, 10.4 kcal/mol (**1**) versus 10.7 kcal/mol (**2**). Overall, these values are in excellent agreement with previously computed reaction barriers using minimum free energy paths with explicit solvent (QM/MM–MFEP method)^[43]^ for the [Ru^II^(tpy)(bpm)(OH_2_)]^2+^ complex.[^44^, ^45^] We note that the best agreement is achieved if corrections for the concentration of the species in solution are included in the computed Gibbs free energies^[46]^ (see uncorrected values in Tables S1 and S2).

**Figure 9:**
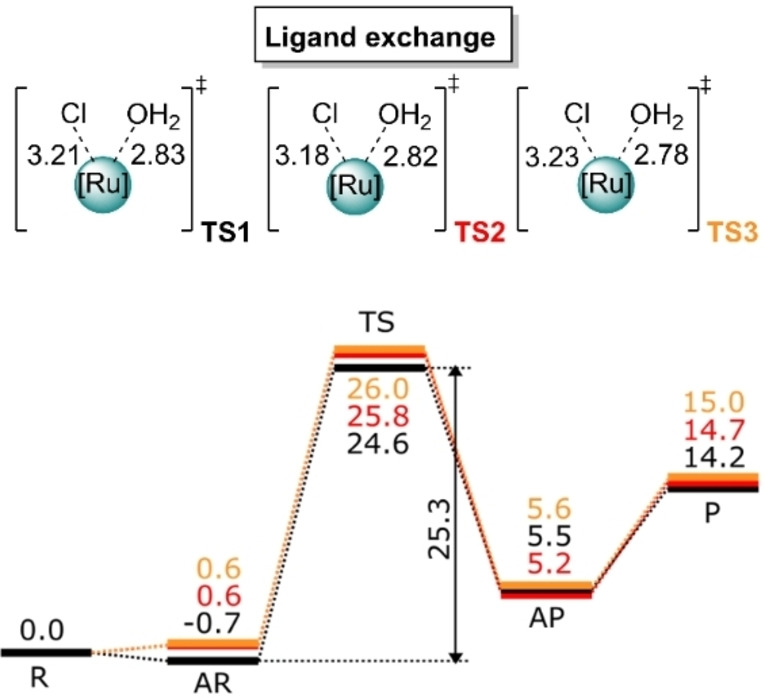
Reaction mechanism and computed relative Gibbs free energies (Δ*G* ${}_{298,H{}_{2}O}$ [kcal/mol]) for the ligand‐exchange step, with corresponding reactants (R), associated reactants (AR), transition states (TS), associated products (AP), and product (P) steps of complexes **1** (black), **2** (red) and **3** (orange). The sum of the reactant energies is taken as a reference (0.0 kcal/mol). Level of theory: UPBE0‐D3BJ‐CPCM(water)/def2‐TZVP//UPBE0‐D3BJ‐CPCM(water)/def2‐SVP.

In any case, our calculations indicate that the WNA step is the rate‐limiting for both complexes, but with nearly identical barriers according to which one would expect similar behavior of the oxygen evolution for both catalysts. Furthermore, if in complex **1** we consider the formation of a stable reactant complex, as found by the calculations (Figure 8a), a higher barrier (so‐called “apparent activation energy”^[47]^) is obtained for **1** (11.6 kcal/mol). These results are at variance with the significantly lower TON observed for complex **2** against **1**, pointing to the essential role of the ligand exchange step over the oxygen evolution. Attempts to obtain the Gibbs free energy path for the water–chlorido ligand exchange for complex **3** have been carried out using well‐tempered meta‐dynamics in a QM/MM setup, with an explicit solvation box. Within feasible simulation times, no dissociation and ligand exchange could be observed. We thus turned to compute the mechanism of the [Ru^II^‐Cl]^+^+H_2_O substitution reaction for the compounds **1, 2** and **3**. As shown in Figure 9, the substitution event is found to be concerted, proceeding via transition states **TS_1,2,3_**. Taking into account also the possible associated reactants and products, the calculated reaction barriers are ΔG≠298,H2O =24.6, 25.8, and 26.0 kcal/mol for **1**–**3**, respectively. The energy differences, despite small, are sufficient to account for the different half‐lives experimentally observed for the considered compounds. Table 3 collects the relative half‐life times obtained from both experiment and theory, using the value of **1** as a reference. One can see that the general trend is satisfactorily reproduced. If for complex **1** we consider the stable associated reactant, which leads to a higher apparent activation energy (25.3 kcal/mol), the qualitative trends of the half‐lives still hold. The trend observed in the reaction barriers can be rationalized looking to the Ru−Cl bond strengths along the different compounds. Indeed, at the equilibrium geometries of the reactants the Ru−Cl distance decreases with the size of the π ligand (**1**: 2.407 Å; **2**: 2.400 Å; **3**: 2.399 Å), which can be correlated with higher barriers. Additionally, we calculated Ru−Cl bond dissociation unrelaxed scans starting from the reactant geometries (Table S4 and Figure S19).

**Table 3:** Comparison of the experimental and computed relative half‐lives (referred to the 0.0 kcal/mol of the reactant), taking the value of **1** as a reference.

System	*t*_1/2_ (X)/*t*_1/2_(1), exp.	*t*_1/2_ (X)/*t*_1/2_(1), calc.
1	1	1
2	5.3	7.5
3	18.1	10.6

Taking the asymptotic limit (5 Å), one can also see that the binding energies are larger with increasing ligand size: 29.8 kcal/mol in complex **1** (bpy), 30.6 kcal/mol in complex **2** (dppz) and 31.1 kcal/mol in complex **3** (tpphz). In conclusion, our calculations demonstrate that the ligand‐exchange step has indeed the largest impact on the experimentally observed TON and the catalytic performance. This result is in line with previous experiments on **1**,^[39]^ in which dioxygen evolution data was recorded independently from the halide‐ and aqua‐ligated complexes, showing lower TON when starting from the halide ligand.

## Conclusion

The influence of π‐conjugation on the substitution of the monodentate Cl ligand in a ruthenium terpyridine water oxidation catalyst has been assessed. Based on ^1^H NMR spectroscopy, the substitution of this monodentate halide ligand is substantially decelerated by the introduction of larger π‐systems, for example, a dppz ligand. The observed effect also alters the catalytic performance of the complexes towards thermal water oxidation with cerium(IV) as the oxidant, due to reduction of the amount of available active aquo‐substituted catalyst during the catalysis. Quantum chemical calculations show that π‐extension in the periphery does not alter the reaction barriers to the water nucleophilic attack or oxygen release steps (comparing **1** and **2**). Instead, the computed Gibbs free energy profiles for the ligand substitution reaction reveal that there is an increase in the energy barrier when dppz is used instead of bpy. The second metal coordination in complex **3** increases this barrier further. The calculated half‐lives of the individual complexes are in accordance with the kinetic data derived from NMR‐spectroscopy and underline the slower ligand exchange in complexes bearing an extended π‐system in the bidentate ligands. We think that this study can give further insight into understanding catalytic performance and builds a foundation for specific optimization when implementing the use of catalysts for more complex architectures, such as polymeric structures.

## Experimental Section

Experimental procedures, information regarding the used measurement setup and additional data for the performed experiments are available in the Supporting Information.

**Computational details**: All structures have been subjected to an unrestricted DFT optimization using the PBE0[^48^, ^49^, ^50^] hybrid functional in combination with the def2‐SVP^[51]^ basis set (def2‐ECP^[52]^ for ruthenium atom) and the D3BJ dispersion correction.[^53^, ^54^] The nature of all stationary points (minima and transition states) was verified through the computation of the vibrational frequencies. The thermal corrections to the Gibbs free energy were combined with the single point energies calculated at the UPBE0‐D3BJ/def2‐TZVP level of theory to yield Gibbs free energies (“*G*_298_”) at 298.15 K. All energies are reported in kcal/mol. The conductor‐like polarizable continuum model CPCM[^55^, ^56^] was applied to consider implicit solvent (water) effects for both geometries and energies. Free energies in solution have been corrected for concentration effects using program GoodVibes.^[57]^ Thereby we set the concentrations to the tabulated value of 55.5 M for water^[46]^ and experimental values of 5 μM and 100 μM at 298.15 K for the solutes involved in the ligand exchange and the water oxidation reactions, respectively.

Sampling of the Gibbs energy surface was attempted with well‐tempered molecular meta‐dynamics^[58]^ and explicit solvent. We employed a periodic QM/MM setup, with the catalyst and a 500 pm solvation shell of water molecules in the QM region and a large solvent box of water molecules (average cell size ca. (7.4 nm)^3^). The QM region used GPW‐DFT^[59]^ with the D3BJ‐corrected^[60]^ PBE^[48]^ functional, DZVP‐MOLOPT‐(SR)‐GTH^[61]^ basis sets and a planewave cutoff of 400 Ry, while the MM water was described by TIP3P water force field.^[62]^ Electronic embedding was employed, using smeared Gaussian charge distributions.^[63]^ The system was equilibrated in an NPT ensemble with 0.5 fs time steps for 50 ps, using a CSVR thermostat^[64]^ and a barostat at 100 kPa, both using 50 fs time constants. Following the equilibration, the time constants of the thermo‐ and barostat were increased to 250 fs and we used well‐tempered molecular meta‐dynamics with ▵*T*=2600 K to force the ligand exchange and sample the Gibbs free energy surface. The Ru−O coordination number and the Ru−Cl coordination number have been chosen as collective variables, and bias potentials with a full‐width‐at‐half‐maximum of 0.05 and a height of 0.5 kJ/mol were added every 25 ps. During the runtime of 30 ps, no ligand dissociation and exchange could be observed.

For the WNA and O_2_ release steps, guesses for geometry optimizations were built inspired in previous publications.[^41^, ^45^] For the ligand exchange reaction, we explored the conformational space of possible associated reactants and products as well as the corresponding transition states of all molecules using the Conformer‐Rotamer Ensemble Sampling Tool (CREST) at the GFN*2*–xTB level of theory.[^65^, ^66^] All DFT calculations have been performed with the Gaussian16‐rev.B.01 program package.^[67]^ All the geometries computed for the WNA, O_2_ release and ligand exchange steps are collected in Tables S5‐S7.

Reaction barriers can be straightforwardly linked to half‐life times using the expression:

$$t_{1/2}=\;\frac{ln2}{k}$$

where *t*_1/2_ is the half‐life and *k* is the rate constant. This rate constant can be obtained from Eyring's equation that read as:

$$k=\frac{\kappa k_{B}T}{h}e^{\frac{{-\Delta G}^{\neq }}{RT}}$$

where Δ*G* ^≠^ is the Gibbs energy of activation, *k*_B_ is the Boltzmann constant, *h* is Planck's constant and *K* is the transmission coefficient, which, according to the so‐called classical formulation of the transition state theory,^[68]^ can be set to 1.

## Conflict of interest

The authors declare no conflict of interest.

## Supporting information

As a service to our authors and readers, this journal provides supporting information supplied by the authors. Such materials are peer reviewed and may be re‐organized for online delivery, but are not copy‐edited or typeset. Technical support issues arising from supporting information (other than missing files) should be addressed to the authors.

Supporting Information
