# Supplementary material for: Strong Ligand Stabilization Based on π‐Extension in a Series of Ruthenium Terpyridine Water Oxidation Catalysts
Source: Chemistry. 2021 Nov 11;27(68):16871–8. doi: 10.1002/chem.202102905 (PMC9299156; doi:10.1002/chem.202102905)
Supplement: Supplementary file 1 — Supporting Information [file CHEM-27-16871-s001.pdf]

Chemistry–A European Journal

Supporting Information

Strong Ligand Stabilization Based on π-Extension in a Series of Ruthenium Terpyridine Water Oxidation Catalysts

Chemistry–A European Journal

Supporting Information

Strong Ligand Stabilization Based on π-Extension in a Series of Ruthenium Terpyridine Water Oxidation Catalysts

Table of contents:

1. Materials and methods S2
2. NMR spectroscopy S5
3. Mass spectrometry S11
4. Cyclic voltammetry S15
5. Photocatalysis S17
6. Computational section S18
7. Author contributions S22
8. References S22

1. Materials and methods:

Materials: 4,4'-di-*tert*-butyl-2,2'-bipyridine, 2,2'-bipyridine and 2,2':6',2''-terpyridine were purchased from ABCR. The synthesis of [Ru(tbbpy)$_2$Cl$_2$] and [Ru(bpy)$_2$Cl$_2$] are literature-known.[1] All solvents used (except for deuterated solvents) were used under air if not stated otherwise.

NMR spectroscopy: ^{1}H (400.13 MHz) NMR spectra were recorded with a Bruker DRX 400 spectrometer. The NMR spectra were recorded in acetonitrile-d$_3$, methanol-d$_4$, acetone-d$_6$ or D_2O at 298 K. ^{1}H-NMR chemical shifts were referenced to the solvent peak for acetonitrile (δ = 1.94 ppm), methanol (δ = 4.87 ppm), acetone (δ = 2.05 ppm) and D_2O (δ = 4.79 ppm). NMR spectroscopic data were analyzed using MestReNova V6.0.2-5475.

Mass spectrometry (MS): MS analysis was performed on a Bruker solariX (2010) Hybrid 7 TFT-ICR for MALDI and with a Bruker Ultraflex III MALDI TOF/TOF for MALDI/TOF measurements. Mass data were converted using Compass DataAnalysisViewer V5.0.

UV-Vis-NIR absorption spectroscopy: UV-vis-NIR spectra were recorded on a JASCO spectrometer V-670. Steady-state UV-Vis spectra were recorded on a single-channel fiber-optic spectrometer (AvaSpec-ULS2048XL), employing a deuterium-halogen light source (AvaLight DH-S-BAL, Avantes Inc., USA).

Electrochemical characterization: Electrochemical measurements were performed using an Autolab potentiostat PGSTAT204 (Metrohm) in three-electrode configuration. Working electrode: glassy carbon disc, diameter 3 mm; counter-electrode: Pt wire; reference electrode: non-aqueous Ag/Ag^+ electrode with 0.01 M $AgNO_3$ in acetonitrile.

Photochemical water oxidation catalysis:[2] all studies were carried out in de-aereated solvents under inert atmosphere. All catalytic experiments were tempered by a custom air cooling setup (25 °C, Fig S1). A screw cap, hermetically sealed vial (diameter: 12.75 ± 0.25 mm, length: 99.00 ± 0.50 mm) equipped with two sensor spots (see "Oxygen detection" below) was used as reaction vessel. The mixtures were stirred during catalysis by a 3D printed custom made stirring bar.

Oxygen detection:[2] Oxygen concentrations were determined using a FireStingO2 optical oxygen meter (Pyroscience, Germany) using oxygen sensitive optical sensor spots (OXSP5, with optical isolation). The spots were glued to the inner glass vessel wall of a screw-capped vial (transparent silicone glue, SPGLUE). The sensor spots are stable between pH 1 – 14, stable in the presence of strong oxidants (the manufacturer suggests cleaning in 3 % aqueous H_2O_2), are not affected by the solvents used (as shown by repeated reproducible catalytic measurements) and are autoclavable at 120 oC. For further information, see the manufacturer information: https://www.pyro-science.com/contactless-fiber-optic-oxygensensor-spots.html. O_2 concentration was measured in µmol/L (solution) and mbar (gas-phase). Both spots were calibrated by two-point calibration: gas-phase calibration was performed against ambient air and Ar-atmosphere. Liquid-phase calibration was performed using a de-oxygenated reaction solution. Solution TONs were calculated based on the detected concentration; gas-phase TONs were calculated via the ideal gas equation.

Calculation of equilibrium constants: Equilibrium constants (K_e) were calculated using equations **1** and **2**:

$$K_e = \frac{k_1}{k_{-1}} \text{ (1)}$$

$$m = -(k_1 + k_{-1}) = -k_{tot} \text{ (2)}$$

Where m is the slope of the linear plot of logarithm of the difference in the integrals of H^a_{Cl} and H^a_{OD} (see Figure 1, main manuscript) derived from the ^{1}H-NMR spectra for compounds **1**, **2** and **3b** *vs.* time.

	**3b**	**1**	**2**
**K_e**	1.33	3.2	2.7
**k_{tot}**	0.054	0.68	0.146
**k_1**	0.031	0.52	0.106
**k_{-1}**	0.023	0.16	0.040

Experimental section:

[Ru(tpy)Cl$_3$]: This compound was synthesized according to a literature-known procedure.[3] $RuCl_3$ x H_2O (0.56 g, 2.1 mmol) and 2,2':6',2''-terpyridine (0.50 g, 2.1 mmol) were added to absolute ethanol (10 mL) and refluxed for 3 h. After evaporation of the solvent in vacuum, the solid was washed well with water and diethyl ether. The product could be isolated as brown solid (80%, 0.74 g) and was used directly for the next steps.

[Ru(tpy)(bpy)Cl]Cl: 2,2'-bipyridine (35 mg, 227 µmol) and [Ru(tpy)Cl$_3$] (100 mg, 227 µmol) were added to dimethylformamide (24 mL) and TEA (2.27 mmol, 315 µL) was added. The mixture was heated in a microwave at 350 W for 2 min and at 300 W for 3 h. After that time, the mixture was poured onto diethyl ether and the precipitate was filtered off. The crude product was purified by slow diffusion of diethyl ether into a methanol solution to yield a purple solid (61%, 78 mg).

^{1}H-NMR (400 MHz, acetone) δ 10.29 (d, *J* = 5.1 Hz, 1H), 8.75 (d, *J* = 8.1 Hz, 2H), 8.62 (d, *J* = 8.2 Hz, 3H), 8.38 (td, *J* = 8.1, 1.4 Hz, 1H), 8.21 (t, *J* = 8.1 Hz, 1H), 8.11 – 8.03 (m, 2H), 7.99 (td, *J* = 8.0, 1.3 Hz, 2H), 7.81 (d, *J* = 6.2 Hz, 3H), 7.55 (d, *J* = 5.6 Hz, 1H), 7.43 – 7.37 (m, 2H), 7.15 – 7.04 (m, 1H).

HRMS (MALDI): $C_{25}H_{19}ClN_5Ru$ calc. 526,03725; found 526.03546 $[M]^+$.

[Ru(tpy)(dppz)Cl]Cl: Dipyrido[3,2-a:2',3'-c]phenazine (dppz) (32 mg, 113 µmol) and [Ru(tpy)Cl$_3$] (50 mg, 113 µmol) were added to dimethylformamide (12 mL) and TEA (1.13 mmol, 155 µL) was added. The mixture was heated in a microwave at 350 W for 2 min and at 300 W for 3 h. After that time, the mixture was poured onto diethyl ether and the precipitate was filtered off. The crude product was purified by slow diffusion of diethyl ether into a methanol solution to yield a purple solid (99%, 77 mg).

^{1}H-NMR (400 MHz, MeOD) δ 10.64 (dd, *J* = 5.3, 1.3 Hz, 1H), 10.08 (dd, *J* = 8.2, 1.4 Hz, 1H), 9.52 (dd, *J* = 8.1, 1.2 Hz, 1H), 8.85 (d, *J* = 8.1 Hz, 2H), 8.73 – 8.66 (m, 3H), 8.64 (dd, *J* = 8.2, 5.4 Hz, 1H), 8.61 – 8.53 (m, 1H), 8.36 (t, *J* = 8.1 Hz, 1H), 8.25 (td, *J* = 7.8, 1.3 Hz, 2H), 8.04 (td, *J* = 8.0, 1.5 Hz, 2H), 7.97 (dd, *J* = 5.5, 1.3 Hz, 1H), 7.91 – 7.78 (m, 2H), 7.67 (dd, *J* = 8.1, 5.5 Hz, 1H), 7.42 – 7.29 (m, 2H).

HRMS (MALDI): $C_{33}H_{21}ClN_7Ru$ calc. 652,05905; found 652.05560 $[M]^+$.

[Ru(tbbpy)$_2$(tpphz)Ru(tpy)Cl](PF$_6$)$_3$: [Ru(tbbpy)$_2$(tpphz)](PF$_6$)$_2$ (20 mg, 15 µmol) and [Ru(tpy)Cl$_3$] (7 mg, 15 µmol) were added to an ethanol water mixture (10 mL, 3/1 v/v) and the mixture was refluxed for 24 h. A color change from red to dark-red/brown could be observed. The solution was allowed to cool down to room temperature and the solvent was evaporated under reduced pressure. Upon addition of an excess of aqueous NH_4PF_6, the precipitated product could be filtered off. The crude product was further purified by slow diffusion of ether to an acetonitrile solution to yield a dark red solid (70%, 20 mg).

^{1}H-NMR (400 MHz, CD_3CN) δ 10.67 (dd, *J* = 5.4, 1.4 Hz, 1H), 10.14 (dd, *J* = 8.2, 1.4 Hz, 1H), 10.04 (dd, *J* = 8.2, 1.3 Hz, 1H), 9.85 (dd, *J* = 8.3, 1.2 Hz, 1H), 9.57 (dd, *J* = 8.2, 1.2 Hz, 1H), 8.57 (ddd, *J* = 8.8, 7.8, 2.0 Hz, 5H), 8.51 (dd, *J* = 6.3, 1.9 Hz, 2H), 8.44 (d, *J* = 8.0 Hz, 2H), 8.27 (dd, *J* = 5.4, 1.3 Hz, 1H), 8.25 – 8.17 (m, 3H), 8.06 (dd, *J* = 8.2, 5.4 Hz, 1H), 7.97 (dd, *J* = 8.3, 5.3 Hz, 1H), 7.94 – 7.85 (m, 3H), 7.75 – 7.66 (m, 4H), 7.63 – 7.53 (m, 4H), 7.53 – 7.46 (m, 2H), 7.25 – 7.21 (m, 2H), 7.21 – 7.14 (m, 2H), 1.46 (d, *J* = 7.2 Hz, 18H), 1.35 (d, *J* = 3.6 Hz, 18H).

HRMS (MALDI): $C_{75}H_{71}ClF_6N_{13}P_2Ru_2$ calc. 1537.33727; found 1537.34021 $[M\text{-}2PF_6]^+$. $C_{75}H_{71}ClF_{12}N_{13}P_2Ru_2$ calc. 1682.30145; found 1682.30455 $[M\text{-}PF_6]^+$.

[Ru(bpy)$_2$(tpphz)Ru(tpy)Cl](PF$_6$)$_3$: [Ru(bpy)$_2$(tpphz)](PF$_6$)$_2$ (50 mg, 60 µmol) and [Ru(tpy)Cl$_3$] (25 mg, 60 µmol) were added to an ethanol water mixture (15 mL, 3 1 v/v) and the mixture was refluxed for 24 h. A color change from red to dark-red/brown could be observed. The solution was allowed to cool down to room temperature and the solvent was evaporated under reduced pressure. Upon addition of an excess of aqueous NH_4PF_6, the precipitated product could be filtered off. The crude product was further purified by slow diffusion of ether to an acetonitrile solution to yield a dark red solid (85%, 82 mg).

^{1}H-NMR (400 MHz, CD_3CN) δ 10.66 (d, *J* = 6.0 Hz, 1H), 10.15 (d, *J* = 9.0 Hz, 1H), 10.07 (d, *J* = 8.2 Hz, 1H), 9.88 (d, *J* = 7.3 Hz, 1H), 9.58 (d, *J* = 7.9 Hz, 1H), 8.65 – 8.49 (m, 7H), 8.43 (d, *J* = 8.0 Hz, 2H), 8.29 (dd, *J* = 15.9, 4.6 Hz, 2H), 8.24 – 8.10 (m, 3H), 8.10 – 8.00 (m, 3H), 7.96 (dd, *J* = 8.3, 5.3 Hz, 1H), 7.94 – 7.84 (m, 5H), 7.80 – 7.72 (m, 2H), 7.72 – 7.67 (m, 2H), 7.56 (dd, *J* = 8.1, 5.5 Hz, 1H), 7.50 (dd, *J* = 13.3, 6.4 Hz, 2H), 7.32 – 7.22 (m, 2H), 7.23 – 7.13 (m, 2H).

HRMS (MALDI): $C_{59}H_{39}ClF_6N_{13}PRu_2$ calc. 1313.08687; found 1313.08609 $[M\text{-}2PF_6]^+$. $C_{59}H_{39}ClF_{12}N_{13}P_2Ru_2$ calc. 1458.05105; found 1458.05040 $[M\text{-}PF_6]^+$.

[Ru(bpy)$_2$(tpphz)Ru(tpy)(C$_3$H$_8$OS)/(H$_2$O)](PF$_6$)$_4$: [Ru(bpy)$_2$(tpphz)Ru(tpy)Cl](PF$_6$)$_3$ (27 mg, 17 µmol) ws dissolved in water and 2-methylthioethanol (72 mg, 84 µmol) was added. The mixture was heated to 60 °C for 15 h under an argon atmosphere. After that time, the complex was precipitated by addition of an excess of NH_4PF_6, filtered and washed with water and diethylether to yield a deep red solid. Due to immediate partial substitution of the methylthioethanol ligand with an aquo ligand, the yield was not determined.

HRMS (MALDI): $C_{62}H_{47}Cl_2N_{13}ORu_2S$ calc. 1295.12113; found 1295.12612.

2. NMR spectroscopy

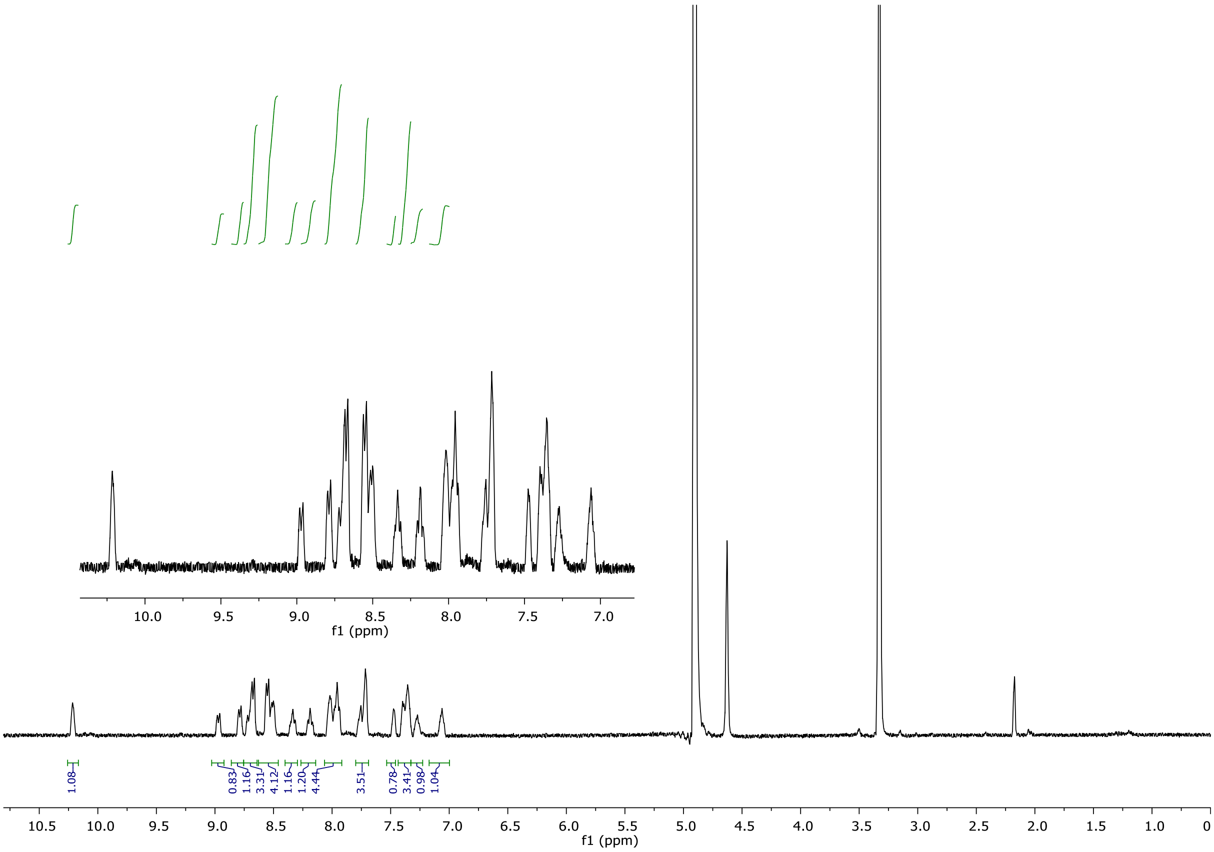

Figure S1: ^{1}H-NMR spectrum of [Ru(tpy)(bpy)Cl]Cl in methanol-d_4 at 25 °C.

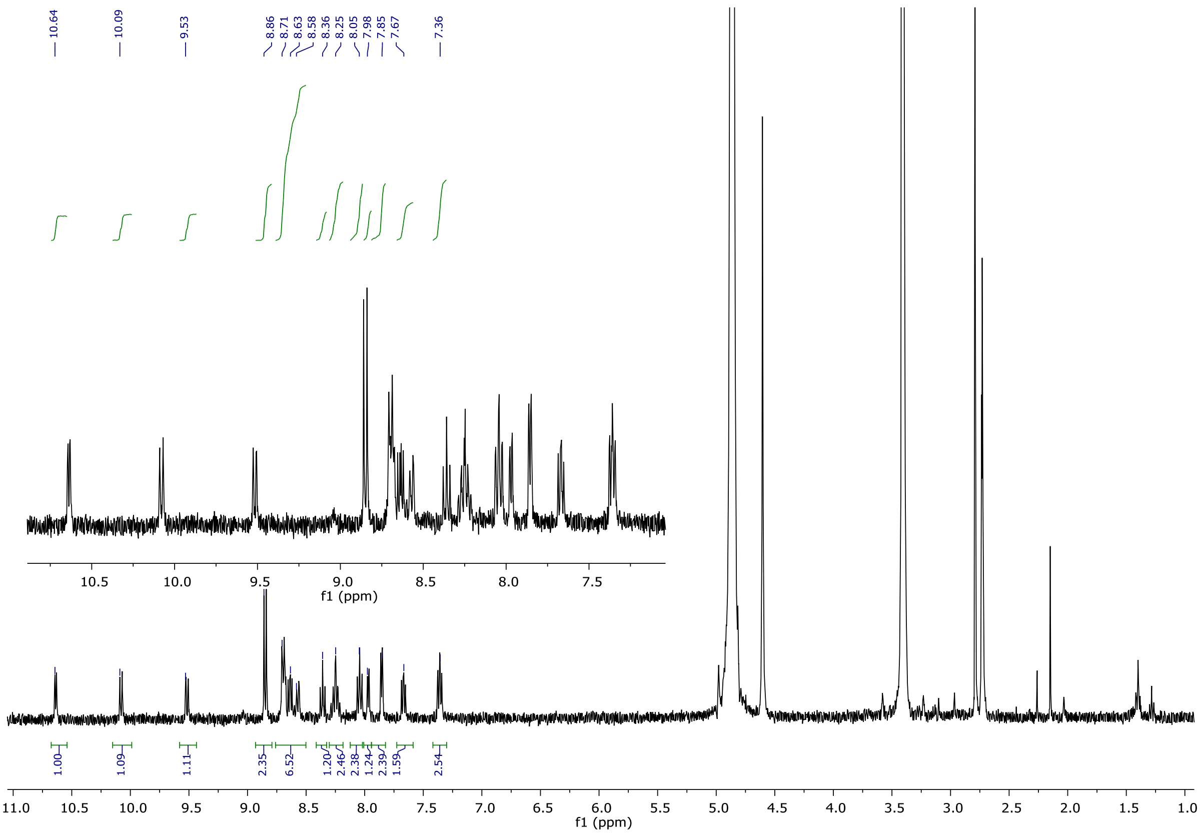

Figure S2: ^{1}H-NMR spectrum of [Ru(tpy)(dppz)Cl]Cl in methanol-d_4 at 25 °C.

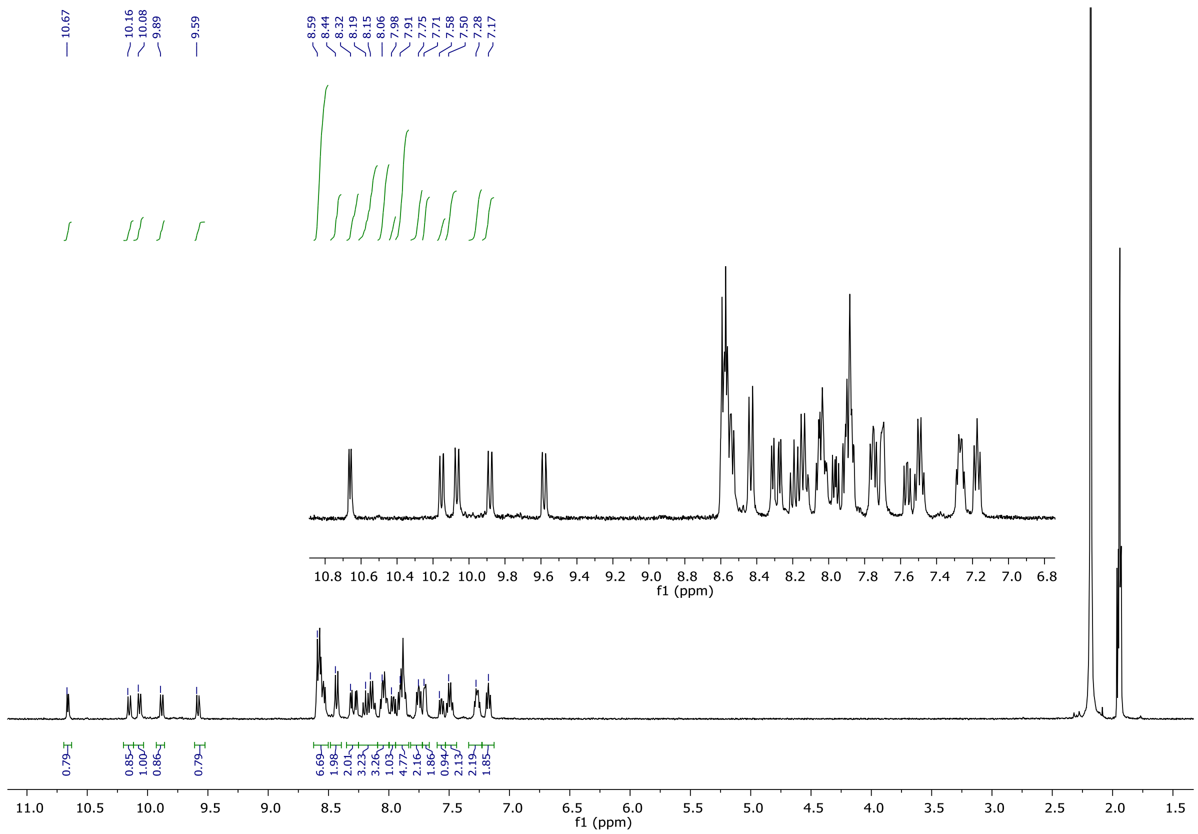

Figure S3: ^{1}H-NMR spectrum of [Ru(bpy)$_2$(tpphz)Ru(tpy)Cl](PF$_6$)$_3$ in acetonitrile-d$_3$ at 25 °C.

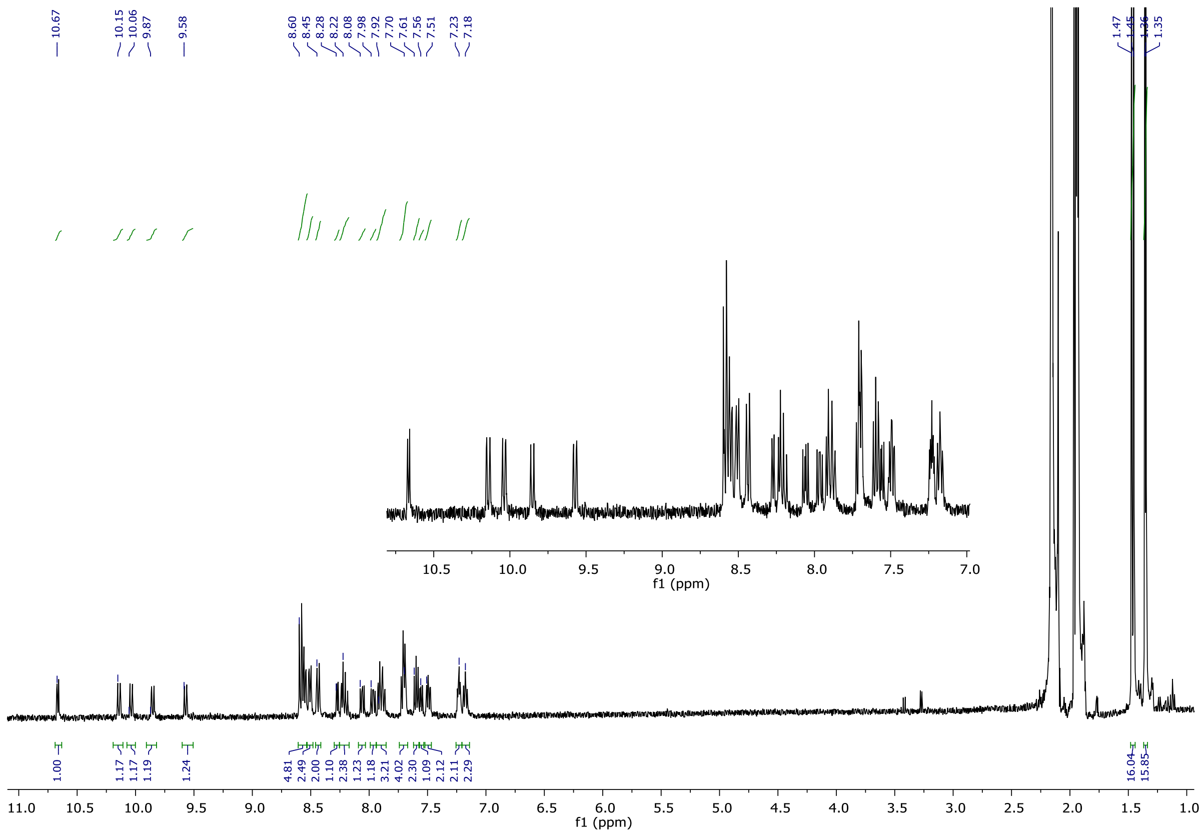

Figure S4: ^{1}H-NMR spectrum of [Ru(tbbpy)$_2$(tpphz)Ru(tpy)Cl](PF$_6$)$_3$ in acetonitrile-d$_3$ at 25 °C.

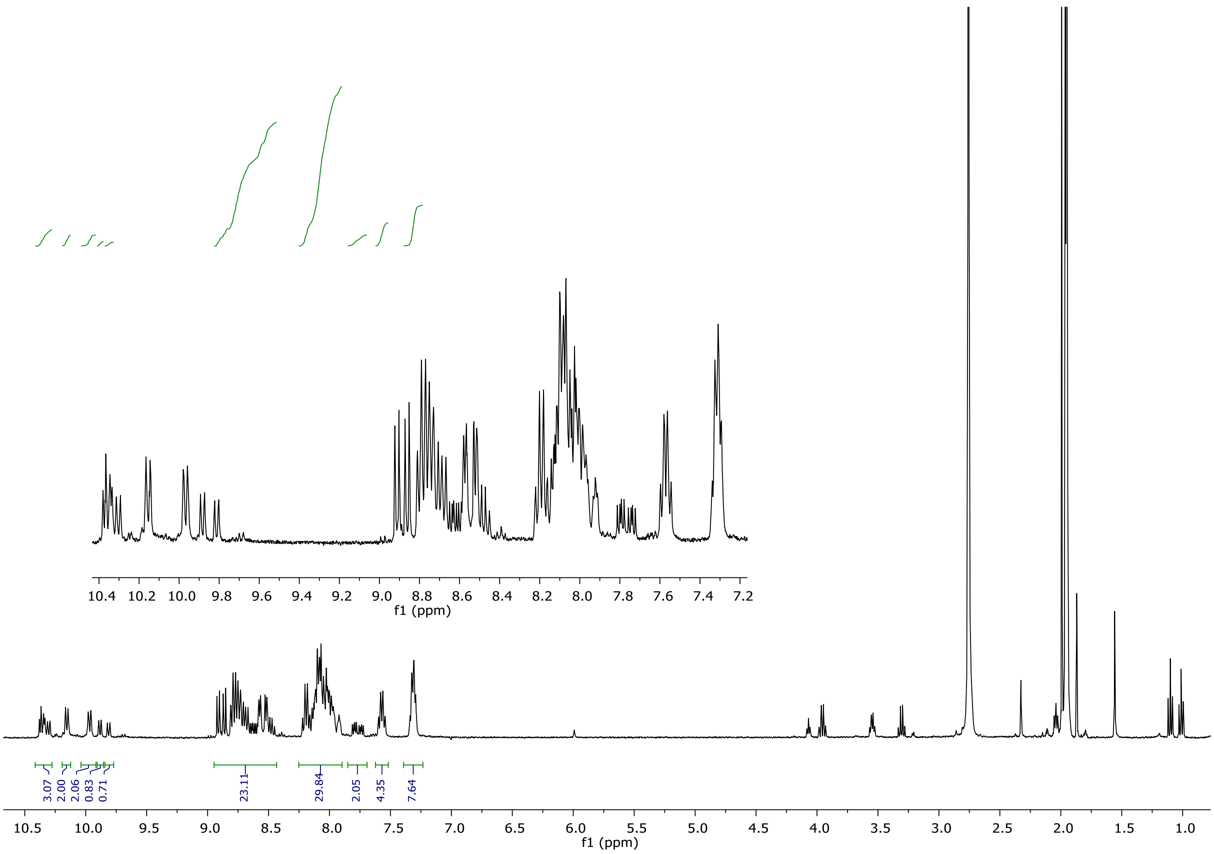

Figure S5: ^{1}H-NMR spectrum of $[Ru(tbbpy)_2(tpphz)Ru(tpy)(C_3H_8OS)/(H_2O)](PF_6)_3$ in acetone-d_6 at 25 °C.

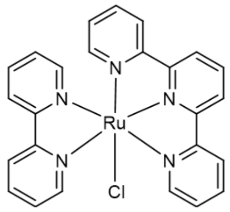

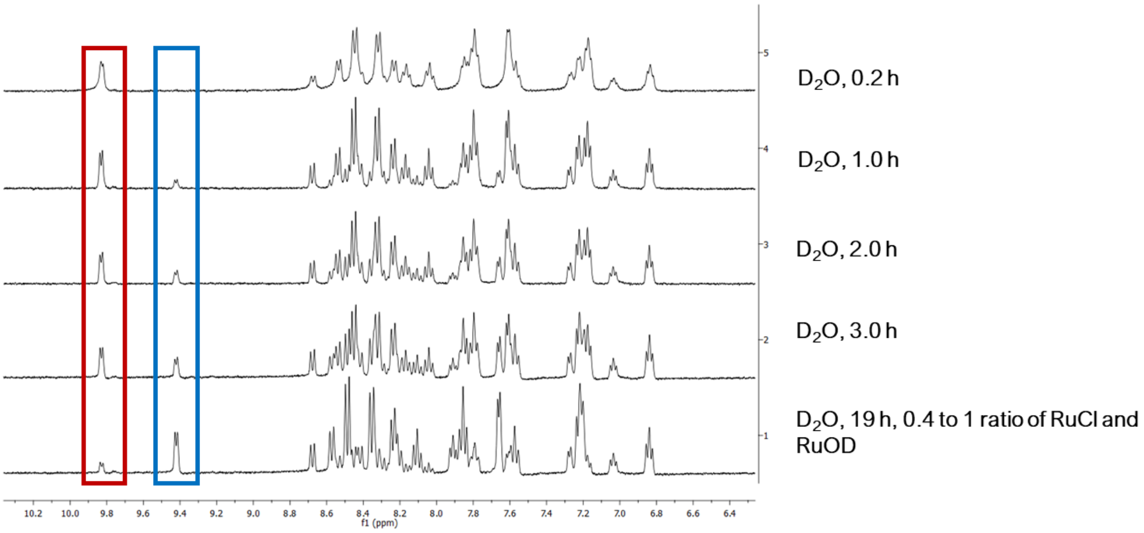

Figure S6: Evolution of the ^{1}H-NMR spectrum of [Ru(tpy)(bpy)Cl]Cl in D_2O vs. time.

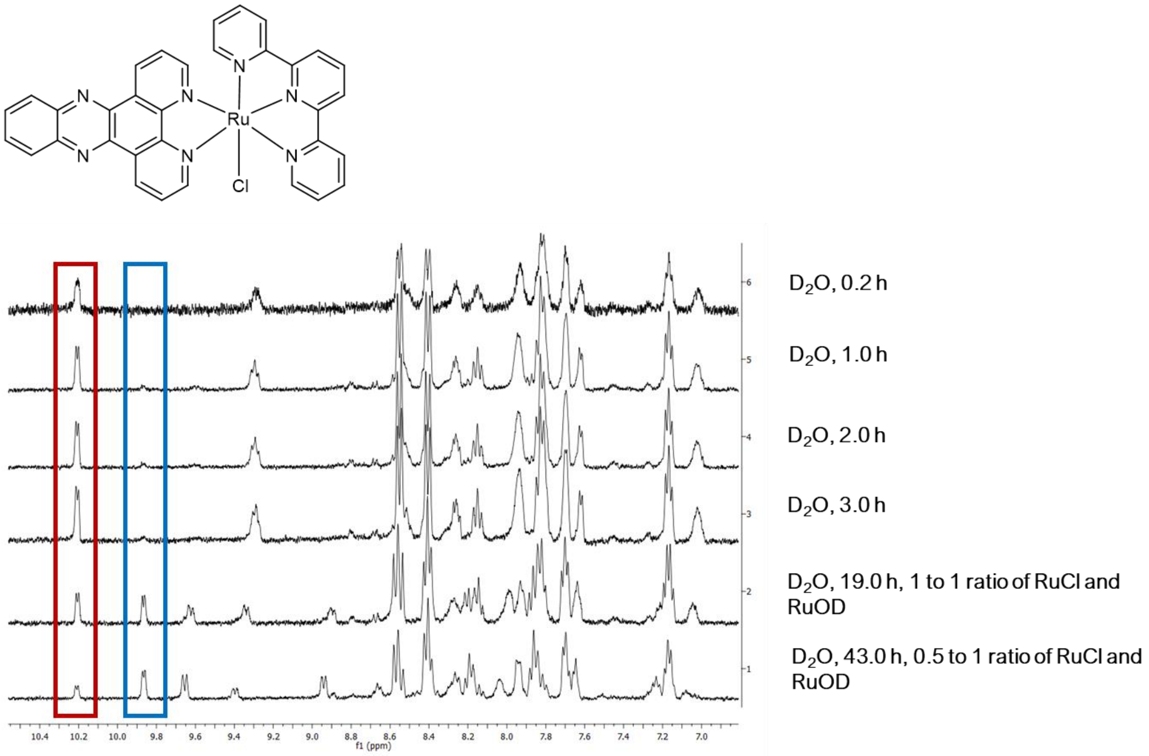

Figure S7: Evolution of the ^{1}H-NMR spectrum of [Ru(tpy)(dppz)Cl]Cl in D_2O vs. time.

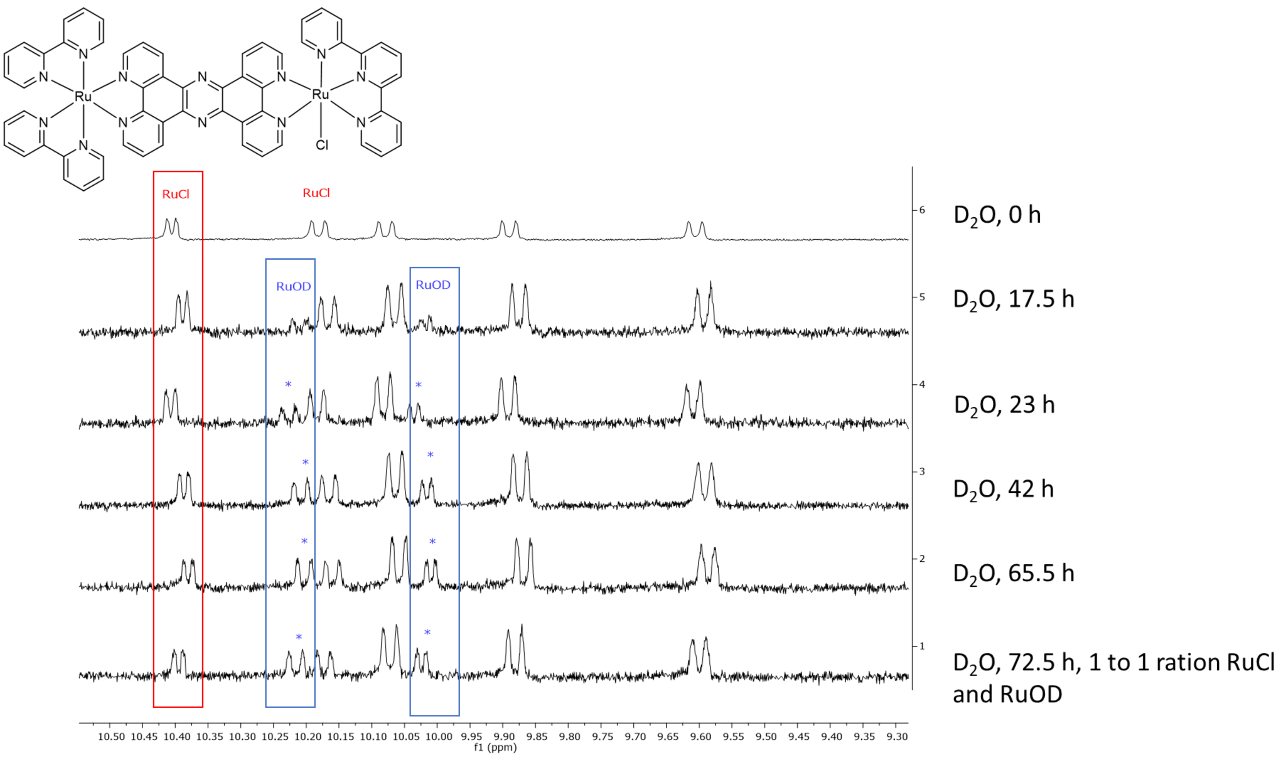

Figure S8: Evolution of the ^{1}H-NMR spectrum of $[Ru(bpy)_2(tpphz)Ru(tpy)Cl]Cl_3$ in D_2O vs. time.

3. Mass spectrometry

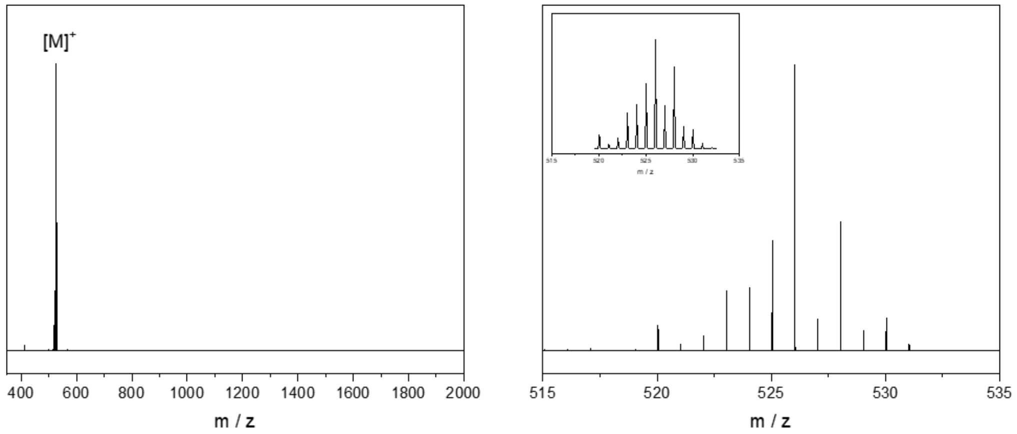

Figure S9: Mass spectrum (MALDI, DCTB) of [Ru(tpy)(bpy)Cl]Cl (left) with magnification and simulation (miniaturization) of the main peak (right).

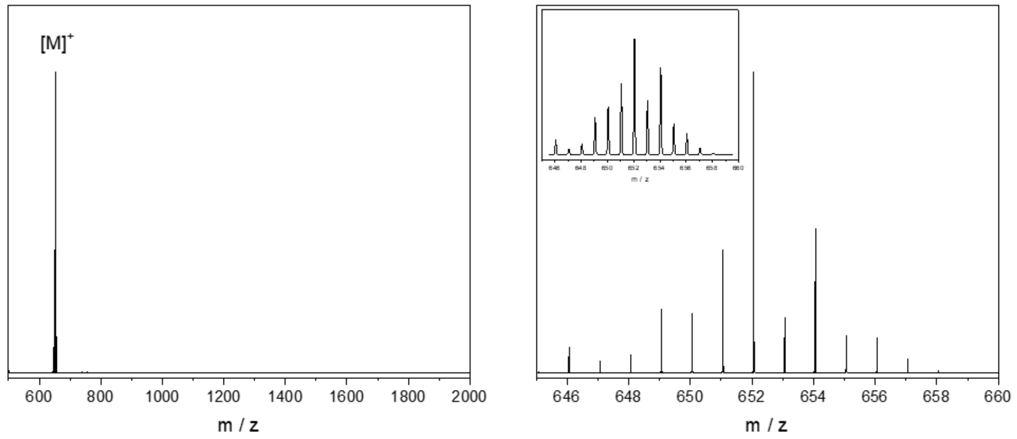

Figure S10: Mass spectrum (MALDI, DCTB) of [Ru(tpy)(dppz)Cl]Cl (left) with magnification and simulation (miniaturization) of the main peak (right).

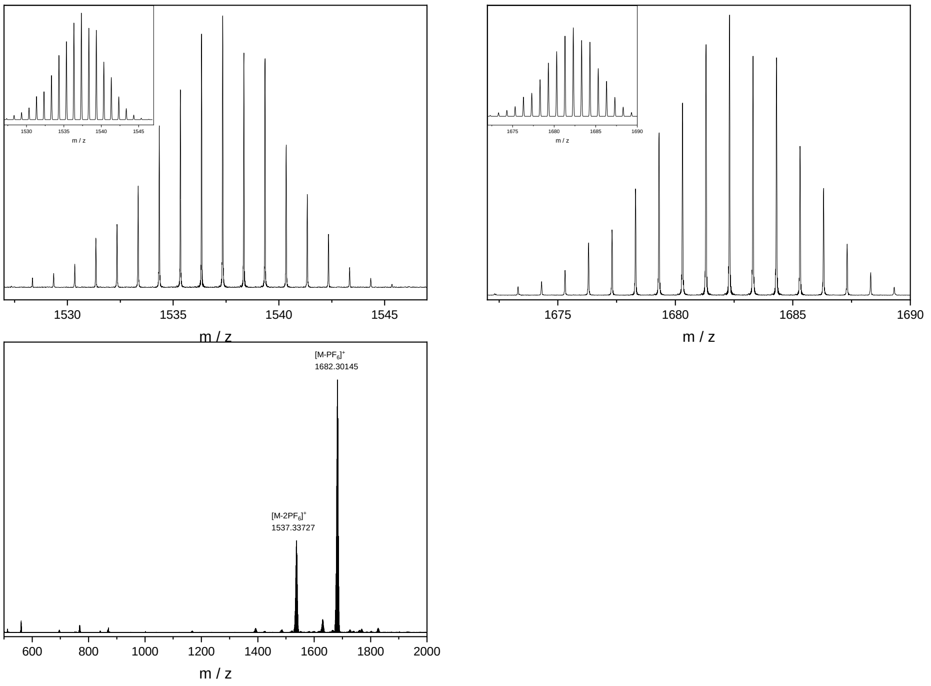

Figure S11: Mass spectrum (MALDI, DCTB) of [Ru(tbbpy)$_2$(tpphz)Ru(tpy)Cl](PF$_6$)$_3$ (bottom) with magnification and simulation (miniaturization) of the main peaks (top).

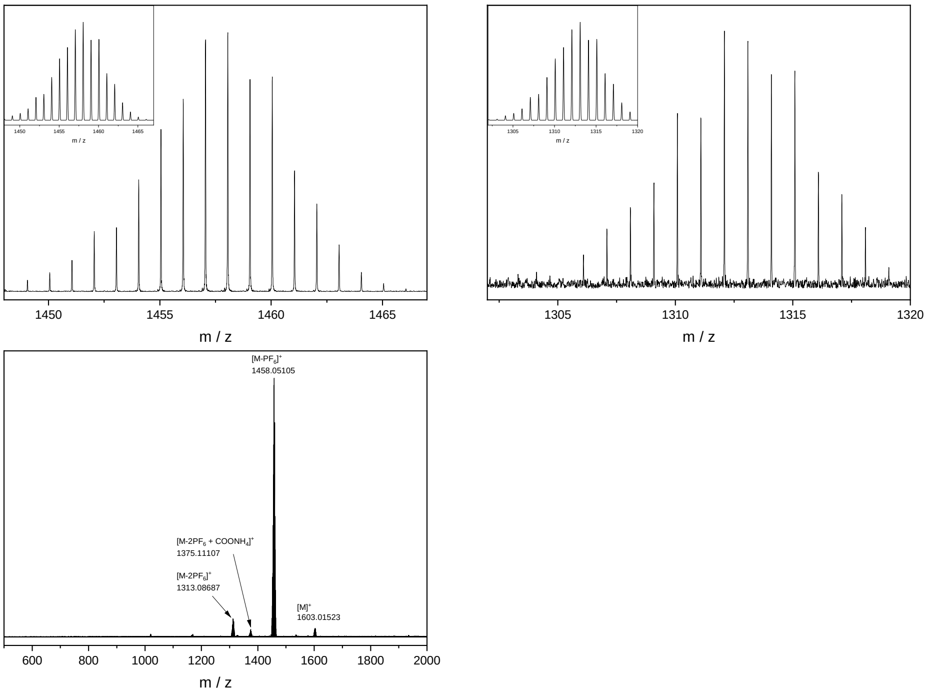

Figure S12: Mass spectrum (MALDI, DCTB) of $[Ru(bpy)_2(tpphz)Ru(tpy)Cl](PF_6)_3$ (bottom) with magnification and simulation (miniaturization) of the main peak (top).

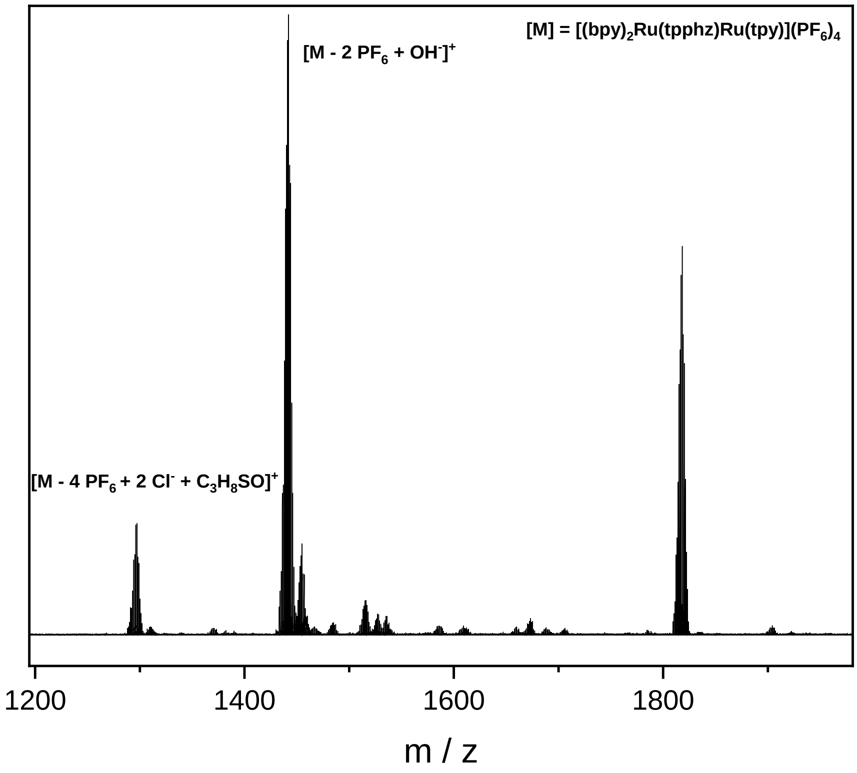

Figure S13: Mass spectrum (MALDI, DCTB) of $[Ru(bpy)_2(tpphz)Ru(tpy)(C_3H_8OS)/(H_2O](PF_6)_3$.

4. Cyclic voltammetry

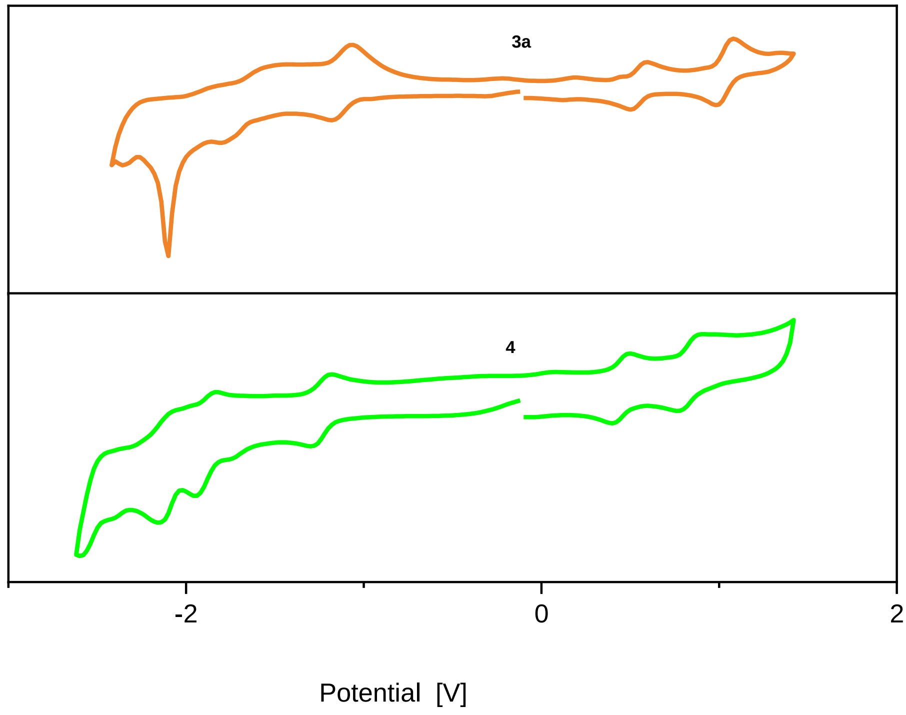

*Figure S14: Cyclic voltammetry of compounds **3a** (orange) and **4** (green) in acetonitrile with TBAPF$_6$ (0.1 M) supporting electrolyte at a scan rate of 100 mV/s at 25 °C. Referenced to Fc/Fc$^+$ couple.*

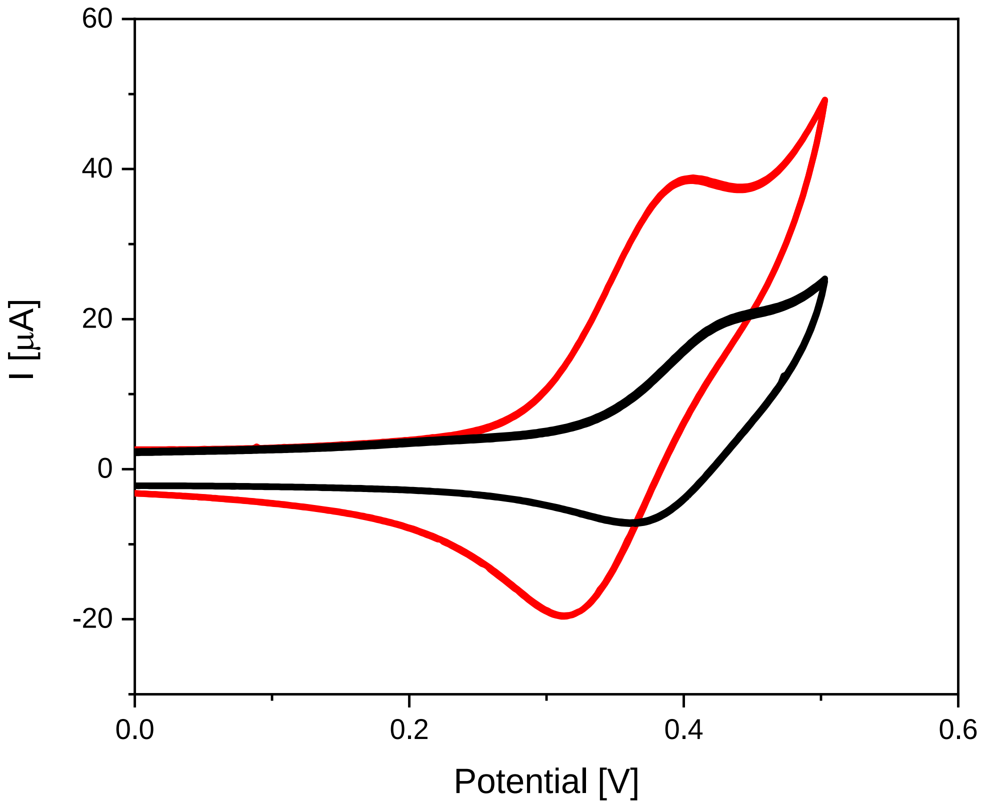

*Figure S15: Cyclic voltammetry of compounds **1** (red) and **2** (black) in acetonitrile with TBAPF$_6$ (0.1 M) supporting electrolyte at a scan rate of 100 mV/s at 25 °C. Referenced to Fc/Fc$^+$ couple.*

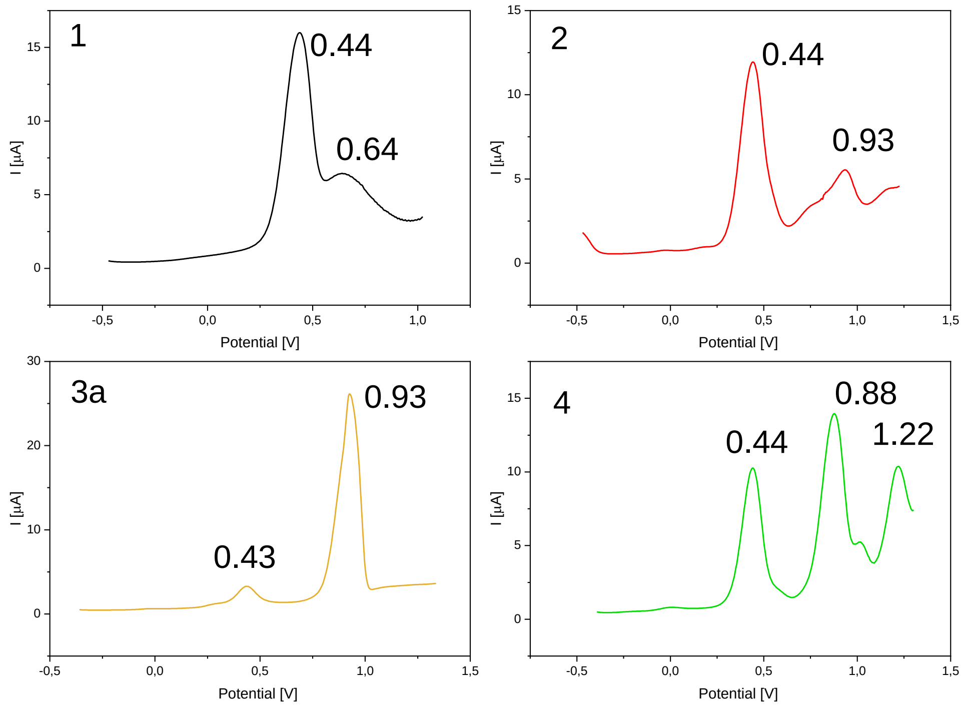

*Figure S16: Differential pulse voltammetry of compounds **1**, **2**, **3a** and **4** in dichloromethane with TBAPF$_6$ (0.1 M) supporting electrolyte at a complex concentration of 1 mM. Potentials are referenced to the Fc$^+$/Fc^0 couple.*

5. Photocatalysis

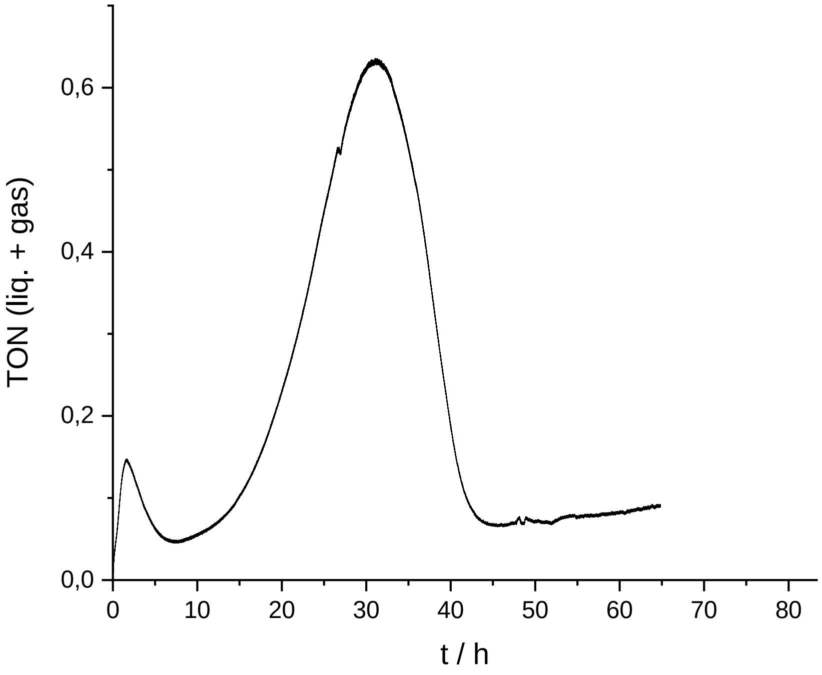

*Figure S17: Photocatalysis using compound **3a** (12 µM) in an aqueous borate buffer (0.08 M, pH 6.92) with $Na_2S_2O_8$ (10 mM) as sacrificial electron donor under blue LED irradiation (λ_{max} = 470 nm, 2 x 50 mW/cm^2).*

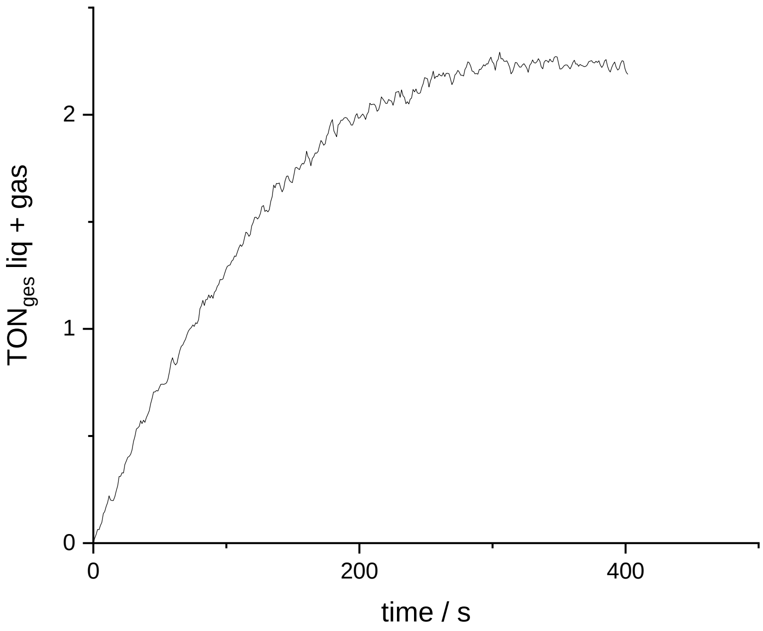

*Figure S18: Photocatalysis using compound **4** (12 µM) in an aqueous borate buffer (0.08 M, pH 6.92) with $Na_2S_2O_8$ (10 mM) as sacrificial electron donor under blue LED irradiation (λ_{max} = 470 nm, 2 x 50 mW/cm^2).*

6. Computational section

	Complex **1**		
	WNA	O_2 release	Ligand exchange
R	0.0 (0.0)	0.0 (0.0)	0.0 (0.0)
AR	-0.7 (7.8)	2.4 (6.7)	-0.7 (3.6)
TS	10.9 (19.4)	10.4 (14.7)	24.6 (28.9)
AP	9.3 (17.8)	-9.7 (-5.4)	5.5 (9.8)
P	14.6 (22.6)	-16.1 (-12.4)	14.2 (17.1)

*Table S1. Computed relative Gibbs free energies ($\Delta G_{298,H2O}$ in kcal/mol) for the water nucleophilic attack (WNA), O_2 release and ligand exchange events, of complex **1**, with corresponding reactants (R), associated reactants (AR), transition states (TS), associated products (AP) and product (P). Within parenthesis, the Gibbs free energies without concentration corrections.* [4]

	Complex **2**		
	WNA	O_2 release	Ligand exchange
R	0.0 (0.0)	0.0 (0.0)	0.0 (0.0)
AR	0.7 (9.3)	2.6 (6.9)	0.6 (4.8)
TS	11.0 (19.6)	10.7 (15.0)	25.8 (30.1)
AP	9.3 (17.8)	-10.8 (-6.6)	5.2 (9.5)
P	14.3 (22.3)	-16.1 (-12.4)	14.7 (17.7)

*Table S2. Computed relative Gibbs free energies ($\Delta G_{298,H2O}$ in kcal/mol) for the water nucleophilic attack (WNA), O_2 release and ligand exchange events, of complex **2**, with corresponding reactants (R), associated reactants (AR), transition states (TS), associated products (AP) and product (P). Within parenthesis, the Gibbs free energies without concentration corrections.* [4]

	Complex **3**
	Ligand exchange
R	0.0 (0.0)
AR	0.6 (4.9)
TS	26.0 (30.3)
AP	15.0 (18.0)
P	5.6 (9.9)

*Table S3. Computed relative Gibbs free energies ($\Delta G_{298,H2O}$ in kcal/mol) for the water nucleophilic attack (WNA), O_2 release and ligand exchange events, of complex **3**, with corresponding reactants (R), associated reactants (AR), transition states (TS), associated products (AP) and product (P). Within parenthesis, the Gibbs free energies without concentration corrections.*[4]

	Complex **1**		Complex **2**		Complex **3**	
Step number	Ru-Cl distance (Å)	Dissociation energies (kcal/mol)	Ru-Cl distance (Å)	Dissociation energies (kcal/mol)	Ru-Cl distance (Å)	Dissociation energies (kcal/mol)
1	2.407	0.0	2.401	0.0	2.399	0.00
2	2.494	0.6	2.487	0.6	-	-
3	2.580	2.1	2.574	2.1	-	-
4	2.667	4.1	2.660	4.2	-	-
5	2.753	6.5	2.746	6.7	-	-
6	2.839	9.0	2.833	9.3	-	-
7	2.926	11.5	2.919	11.9	-	-
8	3.012	14.0	3.006	14.4	-	-
9	3.099	16.3	3.092	16.7	-	-
10	3.185	18.5	3.178	18.9	-	-
11	3.272	20.4	3.265	21.0	-	-
12	3.358	22.0	3.351	22.7	-	-
13	3.444	23.3	3.438	24.1	-	-
14	3.531	24.4	3.524	25.0	-	-
15	3.617	25.0	3.611	25.4	-	-
16	3.704	25.5	3.697	26.0	-	-
17	3.790	25.9	3.783	26.4	-	-
18	3.877	26.5	3.870	26.9	-	-
19	3.963	27.0	3.956	27.4	-	-
20	4.049	27.4	4.043	27.8	-	-
21	4.136	27.8	4.129	28.3	-	-
22	4.222	28.2	4.216	28.6	-	-
23	4.309	28.6	4.302	28.9	-	-
24	4.395	28.8	4.388	29.2	-	-
25	4.482	29.1	4.475	29.5	-	-
26	4.568	29.3	4.561	29.7	-	-
27	4.654	29.5	4.648	29.9	-	-
28	4.741	29.6	4.734	30.1	-	-
29	4.827	29.7	4.821	30.3	-	-
30	4.914	29.7	4.907	30.5	-	-
31	5.000	29.8	4.993	30.6	5.000	31.1

*Table S4. Ru-Cl bond dissociation energy scans for complexes **1**, **2** and **3**, starting from the equilibrium geometry of each complex. In the case of complex **3**, due to the considerably increased size, the relative energy was only evaluated at the limit of 5.0 Å.*

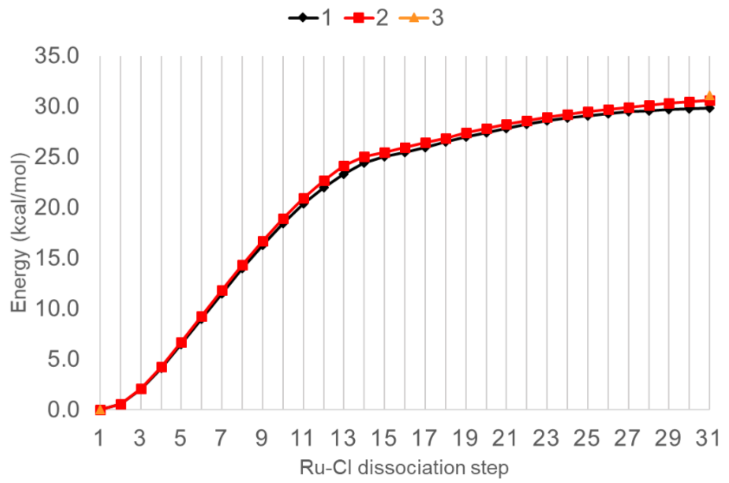

*Figure S19 Plotted values of the Ru-Cl bond dissociation energy scans for complexes **1**,**2** and **3**, from the data depicted in Table S4.*

	R	AR	TS	AP	P
**1**	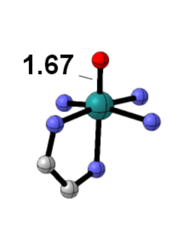	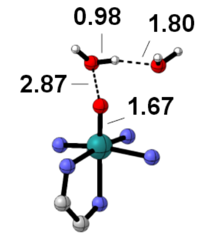	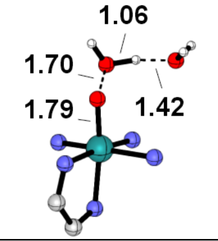	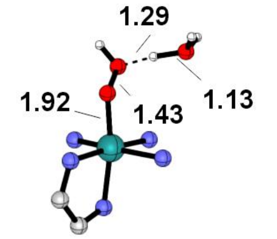	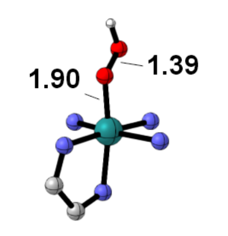
**2**	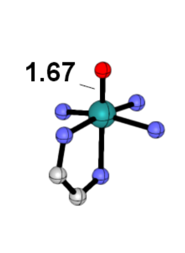	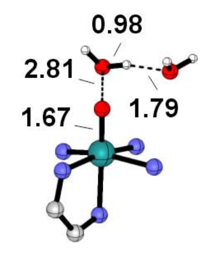	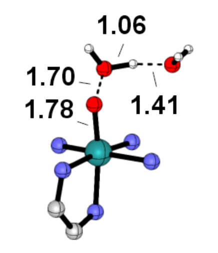	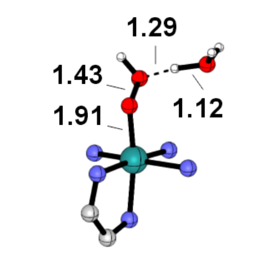	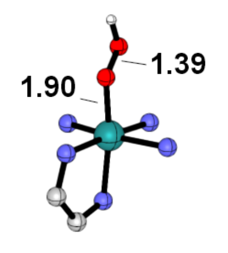

*Table S5 Relevant geometrical changes across the water nucleophilic attack (WNA) step, with corresponding reactants (R), associated reactants (AR), transition states (TS), associated products (AP) and product (P) steps of complex **1** and **2**. The structure of the complexes has been represented in a more simplified manner.*

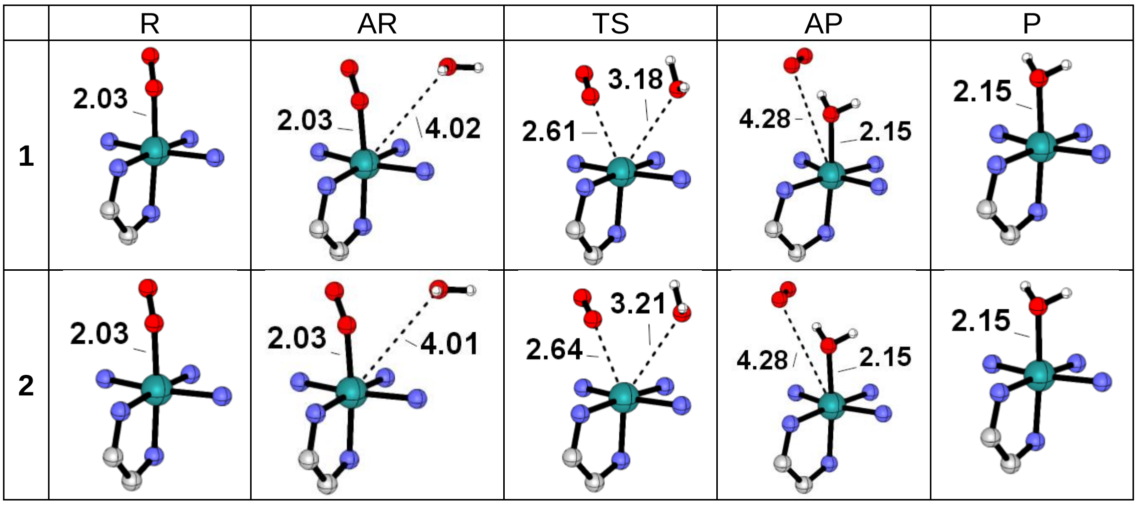

*Table S6 Relevant geometrical changes across the O_2 release step, with corresponding reactants (R), associated reactants (AR), transition states (TS), associated products (AP) and product (P) steps of complex **1** and **2**. The structure of the complexes has been represented in a more simplified manner.*

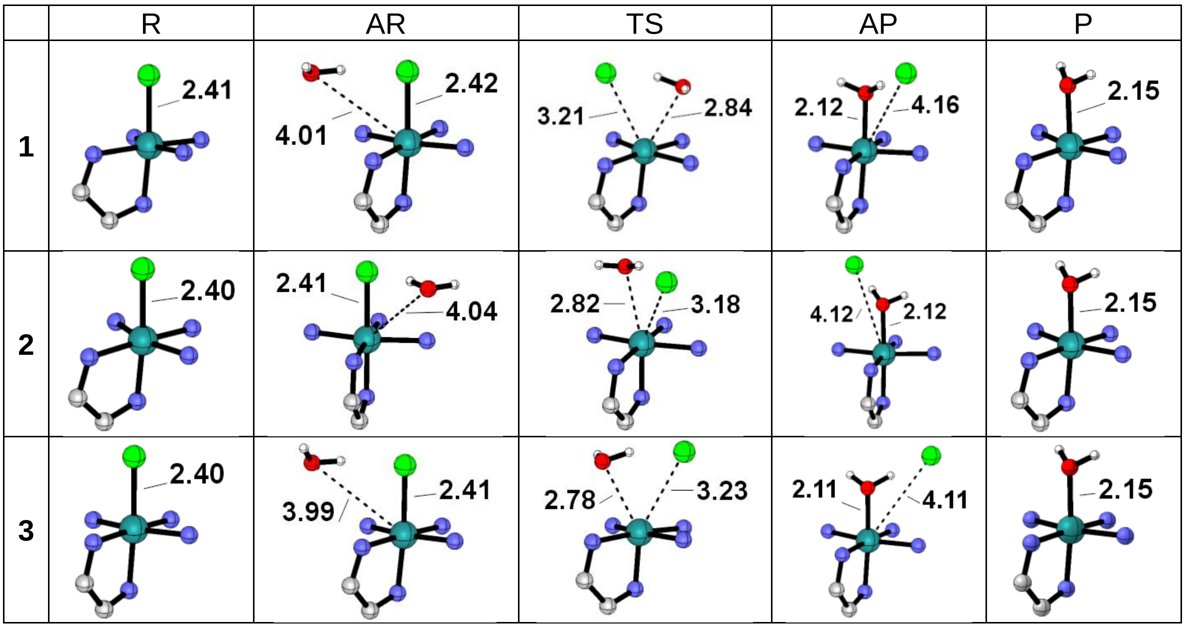

*Table S7 Relevant geometrical changes across the ligand exchange step, with corresponding reactants (R), associated reactants (AR), transition states (TS), associated products (AP) and product (P) steps of complex **1**, **2** and **3**. The structure of the complexes has been represented in a more simplified manner.*

7. Author contributions

SA, SR, LG and SG conceived the study. SA performed and analyzed the experiments, DHC and BM performed and analyzed all DFT calculations and conformational samplings. PS conducted the preliminary well-tempered meta-dynamics simulations. SA and DHC wrote the original draft. LG and SG supervised the theoretical work. SR and AM supervised the experimental work. AM, LG and SR reviewed and edited the manuscript. SR, LG and SG acquired funding. All authors have discussed and approved the manuscript.

8. References

[1] S. Rau, M. Rüben, T. Büttner, C. Temme, S. Dautz, H. Görls, M. Rudolph, D. Walther, A. Brodkorb, M. Duati, C. O’Connor, J. G. Vos, *J. Chem. Soc. Dalt. Trans.* **2000**, 3649–3657.

[2] F. L. Huber, S. Amthor, B. Schwarz, B. Mizaikoff, C. Streb, S. Rau, *Sustain. Energy Fuels* **2018**, 2, 1974–1978.

[3] P. A. Adcock, F. R. Keene, R. S. Smythe, M. R. Snow, *Inorg. Chem.* **1984**, *23*, 2336–2343.

[4] G. Luchini, J. V Alegre-Requena, I. Funes-Ardoiz, R. S. Paton, *F1000Research* **2020**, 9, 1–14.
